# *Jianpi-Huayu-Jiedu* formula improves metabolic dysfunction-associated steatohepatitis through ameliorateing hepatic oxidative stress and lipid accumulation *in vivo* and *in vitro*

**DOI:** 10.3389/fphar.2025.1612726

**Published:** 2025-09-10

**Authors:** Xiaoqin Wu, Tingting Chen, Ziyi Zhang, Danna Wang, Kaili Zhou, Jiayu Li, Like Xu, Cong Meng, Huafeng Pan, Jiawen Huang, Yong Gao, Siyi Li

**Affiliations:** ^1^ State Key Laboratory of Traditional Chinese Medicine Syndrome, Science and Technology Innovation Center, Guangzhou University of Chinese Medicine, Guangzhou, China; ^2^ The Affiliated Traditional Chinese Medicine Hospital, Guangzhou Medical University, Guangzhou, China; ^3^ Department of Traditional Chinese Medicine, People’s Hospital of Longhua, Shenzhen, China

**Keywords:** metabolic dysfunction-associated steatohepatitis (MASH), oxidative stress, lipid accumulation, PI3K/Akt pathway, Jianpi-Huayu-Jiedu formula (JPD), Chinese medicine

## Abstract

Hepatic oxidative stress and lipid accumulation are two key features of metabolic dysfunction-associated steatohepatitis (MASH). Jianpi-Huayu-Jiedu Formula (JPD) has been shown to ameliorate oxidative stress and lipid accumulation, suggesting its potential as an effective treatment for MASH. The current study aimed to explore the therapeutic effects of JPD on MASH. Mice fed a methionine- and choline-deficient (MCD) diet and HepG2 cells induced by oleic acid and palmitic acid (OA&PA) or lipopolysaccharide (LPS) were used as *in vivo* and *in vitro* models, respectively. Network pharmacology, molecular biology, and transcriptomics were employed to investigate the efficacy and mechanisms of JPD. Network pharmacology analysis indicated that the PI3K-AKT signaling pathway is a potential target of JPD in MASH. Molecular docking also revealed the binding of active components to AKT1 or PPARG. *In vitro*, JPD significantly reduced LPS-induced inflammation and OA&PA-induced lipid accumulation. *In vivo*, JPD inhibited MCD-induced liver injury, inflammation, oxidative stress, and lipid accumulation. Transcriptomic analysis further suggested that the PI3K/AKT and PPAR signaling pathways are involved. An AKT inhibitor significantly attenuated the protective effect of JPD. JPD ameliorates hepatic oxidative stress and lipid accumulation by activating both the PI3K/AKT and PPAR pathways. Our study provides a novel strategy for treating MASH with traditional Chinese medicine.

## 1 Introduction

Metabolic dysfunction-associated steatohepatitis (MASH) is a progressive liver disorder distinguished by excessive lipid accumulation, chronic inflammation, and hepatocellular damage, prominently manifested by hepatocyte ballooning. This pathological condition is often accompanied by varying extents of fibrosis and has risen to prominence as the most prevalent chronic hepatic disease worldwide (Younossi et al., 2018; Harrison et al., 2023). The incidence of MASH continues to escalate, positioning it as a principal driver of end-stage liver failure and a major indication for liver transplantation. Although substantial progress has been made in delineating the pathogenesis of MASH and in the identification of novel molecular targets, the number of pharmacotherapies that have obtained regulatory approval remains limited (Xu et al., 2022a).

The progression of MASH is closely related to a series of liver injuries. These injuries are driven by several factors, including lipotoxicity, oxidative stress, and redox imbalance, which is characterized by excessive nitric oxide production. Other contributing factors are endoplasmic reticulum stress, inflammation, and apoptosis. These processes sequentially affect different groups of liver cells. Over time, these pathological events culminate in the activation of liver regeneration and fibrogenesis, leading to excessive collagen synthesis and extracellular matrix accumulation, ultimately accelerating the progression of hepatic fibrosis and cirrhosis (Ding et al., 2021; Lee et al., 2022). Elevated intracellular levels of reactive oxygen species (ROS) instigate oxidative modifications of cellular macromolecules, inflicting damage on lipids, proteins, and nucleic acids, thereby triggering cellular injury, inflammatory responses, and fibrotic remodeling (Chen et al., 2020; Gao et al., 2022; Zhao et al., 2020a). Consequently, similar to other chronic hepatic disorders, persistent liver inflammation likely precedes the onset of MASH and serves as a pivotal pathophysiological driver of hepatic fibrogenesis (Filozof et al., 2017).

Lipotoxicity represents a pivotal pathological process in the progression of MASH in affected individuals (Zambo et al., 2013). Specifically, during excessive hepatic lipid accumulation, both the composition of the accumulated fats and the cellular mechanisms employed by hepatocytes to manage this metabolic burden determine whether the liver undergoes adaptive changes leading to benign hepatic steatosis or experiences cellular demise through diverse molecular pathways (Arguello et al., 2015). In the latter scenario, hepatocytes release stress-associated molecular patterns, often referred to as danger signals, which initiate the activation of sterile inflammatory pathways—those occurring in the absence of pathogenic infection. If persistently activated, these inflammatory cascades drive chronic hepatic injury, dysregulated tissue repair, and fibrosis development (Seki and Schwabe, 2015). Therefore, abnormal lipid accumulation can promote disease progression.Additionally, Lipid oxidation is a critical metabolic process that maintains cellular energy homeostasis and lipid balance. Mitochondrial β-oxidation and peroxisomal fatty acid oxidation are two key pathways that contribute to fatty acid catabolism. Impaired mitochondrial β-oxidation and peroxisomal fatty acid oxidation (FAO) jointly disrupt hepatic lipid homeostasis, driving MASH progression. Mitochondrial β-oxidation, mediated by carnitine palmitoyltransferase 1A (CPT1A), is essential for long-chain fatty acid catabolism; its suppression leads to toxic lipid accumulation (e.g., diacylglycerols) and reduces ATP synthesis, exacerbating hepatocyte injury (Rinella et al., 2023). Concurrently, peroxisomal FAO deficiency—marked by acyl-CoA oxidase 1 (ACOX1) dysfunction causes very-long-chain fatty acid (VLCFA) retention and excessive H_2_O_2_ production, which activates NLRP3 inflammasomes and promotes fibrogenesis (Friedman et al., 2018). This dual impairment creates a vicious cycle: mitochondrial dysfunction limits peroxisomal metabolite clearance, while peroxisomal oxidative stress further damages mitochondria. These pathways are interconnected, with peroxisomal β-oxidation acting as a sensor for intracellular fatty acid levels and influencing mitochondrial function (Mah et al., 2024). Dysregulation of these processes is implicated in metabolic disorders such as hepatic steatosis and insulin resistance (Yao et al., 2023). In MASH, understanding their roles is critical to decode the disrupted lipid metabolism network and reveal precision therapeutic targets.

The multi-faceted therapeutic effects of Chinese Materia Medica (CMM) in combating MASH, which arises from a complex interplay of multiple pathogenic factors, have demonstrated substantial efficacy. Its pharmacological actions are characterized by a multi-component, multi-target, and multi-pathway approach, enabling comprehensive modulation of disease-related molecular mechanisms and contributing to its promising therapeutic potential (Chen et al., 2021). Based on our previous research, it has been found that the traditional Chinese medicine Decoction-Jianpi Huayu Jiedu Formula (JPD) has pharmacological effects in reducing inflammation, oxidative stress, and other related conditions (Tian et al., 2021). Previous studies have indicated that Astragalus membranaceus (Huangqi) and Atractylodes macrocephala (Baizhu), as key pharmacologically active constituents of JPD, possess notable therapeutic potential in the management of metabolic dysfunction-associated steatohepatitis (MASH) (Chen et al., 2024; Liu et al., 2019). Also, further study has verified its regulation in PI3K/AKT signaling pathway (Xie et al., 2021). A study showed that NRF2 activation through the PI3K/AKT signalling pathway significantly enhances hepatocellular anti-oxidant capacity and relieves mitochondrial dysfunction by inhibiting NOX2 activation in mice fed an HFD, suggesting that PI3 K/AKT/NRF2 signal transduction plays a role in the regulation of hepatocellular oxidative damage (Liao et al., 2019). AKT activation leads to inhibition of nuclear factor kappa B (NF-κB), a transcription factor crucial in inflammation and fibrogеnеsis (Liu et al., 2022), and its inhibition may be companied by a reducing in pro-inflammatory cytokines, such interleukin-6 (IL-6) and tumor necrosis factor- alpha (TNF-α) levels (Xia and Hui, 2014), while anti-inflammatory cytokines like interleukin-10 (IL-10) are increased. Activation of the PI3K/AKT pathway not only promotes cell survival and inhibits apoptosis but also amplifies NRF2-mediated antioxidant responses (Xuanfei et al., 2017). Furthermore, pharmacological modulation of PI3K/AKT signaling augments NRF2 activity and alleviates liver inflammation in experimental models (Zhou et al., 2022). Therefore, given its unique pharmacological properties, JPD holds promise as a potential therapeutic approach for managing MASH. This study aimed to investigate the possible therapeutic efficacy of JPD and elucidate its underlying molecular mechanisms in the context of MASH treatment.

## 2 Materials and methods

### 2.1 Main reagents

Silymarin (Batch No.: I2109501) was supplied by Shanghai Aladdin Biochemical Technology Co., Ltd. MCD was sourced from Trophic Animal Feed High-Tech Co., Ltd., China. ALT (Cat. No.: C009-2-1), AST (Cat. No.: C010-2-1), TC (Cat. No.: A111-1-1), and TG (Cat. No.: A110-1-1) were purchased from Nanjing Jiancheng Bioengineering Institute. Lipopolysaccharide (LPS) was supplied by Sigma-Aldrich Co. (Shanghai, China). The Cell Counting Kit-8 (CCK-8) was purchased from Dojindo Laboratories (Kumamoto, Japan). Fetal bovine serum (FBS) and DMEM medium were provided by Biological Industries (Kibbutz Beit Haemek, Israel). AKT1 inhibitor (MK-2206 2HCL) was purchased from Shanghai Macklin Biochemical Technology Co., Ltd (Batch No.:C16883970).

### 2.2 Composition and preparation of JPD

Panaxnotoginseng (Chinese name: San Qi), Pseudostellaria heterophylla (Chinese name: Tai Zi Shen), Curcuma phaeocaulis Valeton (Chinese name: E Zhu), Hericium erinaceus (Chinese name: Hou Tou Gu), Poria cocos (Chinese name: Fu Ling), Astragalus membranaceus (Chinese name: Huang Qi), Scleromitrion diffusum (Chinese name: Bai Hua She She Cao), Atractylodes macrocephala Koidz. (Chinese name: Bai Zhu), Gekko (Chinese name: Shou Gong), All herbs were obtained from the Laboratory of the Scientific Innovation Center at Guangzhou University of Chinese Medicine. Herbs were mixed with the proportion of 5:10:9:16:10:20:15:10:5. JPD extraction was prepared following previous protocol. In brief, Shou Gong and San Qi were grind into fine medicinal powder, the volatile oil of E Zhu was extracted. Then, the residual crude medicinal herbs were immersed in purified water at a ratio of 1:10 (w/v) for 30 min, followed by decoction for 1.5 h under controlled boiling conditions. Finally, the extraction was filtered, concentrated, freeze-dried, and mixed with the fine powder and volatile oil mentioned above.

### 2.3 Identification of major components of JPD by UHPLC-QTOF-MS

The chromatographic separation and mass spectrometric detection were performed using an ultra-high-performance liquid chromatography system (1,290 Infinity II, Agilent Technologies) interfaced with a quadrupole time-of-flight mass spectrometer (6545XT, Agilent Technologies). Separation was achieved on a reversed-phase C18 column (ZORBAX Eclipse Plus, 2.1 × 50 mm, 1.8 μm) maintained at 35 °C. A binary mobile phase system consisting of (A) 0.1% formic acid in water and (B) 0.1% formic acid in acetonitrile was employed with the following gradient program: initial 5% B (0–0.5 min), 5%–10% B (0.5–3 min), 10%–30% B (3–10 min), 30%–60% B (10–15 min), 60%–100% B (15–18 min), isocratic 100% B (18–21 min), followed by re-equilibration to 5% B at 21.1 min (flow rate: 0.35 mL/min; injection volume: 2 μL). For mass spectrometric analysis, the electrospray ionization source was operated in positive mode with the following parameters: drying gas (320 °C, 10 L/min), sheath gas (400 °C, 12 L/min), nebulizer pressure (45 psi), capillary voltage (4000 V), and nozzle voltage (500 V). The fragmentor voltage and collision energy were set to 180 V and 30 eV, respectively. Full-scan mass spectra (m/z 100–1700) were acquired in centroid mode, and subsequent data processing was conducted using MassHunter Qualitative Analysis software (version B.10, Agilent Technologies). Compound identification was achieved through systematic analysis of retention times, high-resolution molecular masses, and MS/MS fragmentation patterns, with validation against the TCM-PCDL database, PubChem, and relevant literature. Our analysis revealed eight predominant bioactive constituents: (1) quercetin, (2) berberine, (3) pseudoginsenoside F11, (4) ginsenoside Rb1, (5) isoallylbenzene, (6) phthalic anhydride, (7) purine, and (8) phenethylamine, along with other pharmacologically relevant compounds. Positive ion mode mass spectrometry facilitated the detection and characterization of quercetin, berberine, pseudoginsenoside F11, isoallylbenzene, phthalic anhydride, and phenethylamine, while negative ion mode proved optimal for identifying pseudoginsenoside F11, ginsenoside Rb1, and purine. Detailed analytical results are presented in Supplementary Material 2.

### 2.4 Network pharmacology prediction

The TCMSP database (https://tcmspw.com/tcmsp.php) was utilized to identify the potential bioactive compounds present in JPD and their corresponding molecular targets (Ru et al., 2014). Two drug screening criteria were applied: (1) oral bioavailability threshold ≥30%; (2) drug-likeness threshold ≥0.18. The GeneCardsdatabase (https://www.genecards.org) was employed to identify potential molecular targets associated with MASH. A Venn diagram was generated using the Venny tool, while the Cytoscape 3.7.0 software was utilized to construct a network mapping the interactions between JPD, its targets, and MASH-related pathways. The STRING database (https://cn.string-db.org/) was used to analyze the overlapping proteins between JPD and MASH (Szklarczyk et al., 2017). The Cytoscape 3.7.0 software was used to construct a protein-protein interaction network (PPI). Kyoto Encyclopedia of Genes and Genomes (KEGG) pathways was performed using the DAVID platform (https://david.ncifcrf.gov) (Huang et al., 2009).

### 2.5 Molecular docking

Molecular docking studies were carried out to evaluate the binding affinities and interaction mechanisms between the potential bioactive compounds and their potential molecular targets. The chemical structures of the compounds were sourced from the PubChem compound database, while the three-dimensional structures of AKT1, PPARα, and NRF2 were retrieved from the Protein Data Bank (PDB). Following the removal of water molecules and the addition of polar hydrogen atoms to the protein structures, docking simulations were conducted using AutoDock Vina version 1.2.2. The binding energy was employed to quantify the interaction strength between the ligand molecules and their respective receptor proteins. In general, a docking free energy value lower than −4.25 kcal/mol signifies a moderate binding affinity, while a value below −5.0 kcal/mol indicates a strong and favorable binding interaction between the ligand and receptor.

### 2.6 Cell culture and pretreatment

The HepG2 cells were cultured in Dulbecco’s Modified Eagle Medium (DMEM) supplemented with 10% fetal bovine serum, 100 U/mL penicillin, and 0.1 mg/mL streptomycin. Following cellular adhesion, the cells were treated with oleic acid (OA) at a concentration of 660 μmol/L and palmitic acid (PA) at 330 μmol/L for 24 h to establish a lipid metabolism disturbance model. Alternatively, cells were exposed to lipopolysaccharide (LPS) at 1 μg/mL to induce an inflammation model. In the control group, cells were cultured in regular medium, while in the model groups, the cells were treated as described above. For the treatment groups, interventions with 25 μg/mL, 50 μg/mL, and 100 μg/mL were applied for 24 h, followed by sample collection for subsequent analysis. In the inhibitor experiments, the cells were subjected to a 24-h pre-treatment with the specified inhibitors (MK-2206 2HCL) prior to the administration of the drug.

### 2.7 Cell viability assay

To evaluate the impact of JPD, which is known for its properties of tonifying the spleen and promoting blood circulation, on cell viability, 2 × 10^4^ cells per well were seeded into a 96-well plate. After 24 h, the cells were exposed to different concentrations of JPD for an additional 24 h, while the control group was treated with an equivalent volume of complete culture medium. Subsequently, cell viability was determined following the protocol of the CCK-8 assay.

### 2.8 Animals

C57BL/6J mice (8 weeks old, weighing 18–22 g) were sourced from the Guangdong Medical Laboratory Animal Center (Foshan, China) and kept in a controlled laboratory environment, with a 12-h light/dark cycle and *ad libitum* access to food and water. Mice were devided into five groups, the control group (Ctrl), the model group (MCD), the positive control group (MCD + Silymarin), the low-dose drug group (MCD + JPD-L), and the high-dose drug group (MCD + JPD-H). Mice in Ctrl group received a normal diet, while other mice fed with MCD for 4 weeks. During this period, mice in Ctrl and MCD received gastric gavage of saline, once daily for 4 weeks. Mice in the MCD + Silymarin group received silymarin (100 mg/kg), and mice in MCD + JPD-L and MCD + JPD-H received JPD 1.25 g/kg and 2.5 g/kg, respectively. All aspects of animal care and experimental procedures were conducted in accordance with the ethical guidelines set forth by the Animal Ethics Committee of Guangzhou University of Chinese Medicine, and the protocols were subsequently approved by the committee (Approval No.: 20230120001).

### 2.9 Histopathological examination, immunofluorescence evaluation, and staining of lipid droplets

For histopathological analysis, liver tissues were initially fixed in 4% paraformaldehyde for 24 h. After fixation, the tissues were dehydrated, embedded in paraffin, and sectioned for routine hematoxylin-eosin staining (H&E) staining. A separate segment of the hepatic tissues was placed in a 30% sucrose solution for 48 h. After dehydration, the tissues were embedded in an OCT compound and cut into 5-μm thick sections. These sections were subsequently rinsed with 60% isopropanol and stained using Oil Red O following the protocol provided by the manufacturer. For immunofluorescence analysis, liver tissue sections were incubated overnight at 4 °C with primary antibodies targeting F4/80 and CD11b (both sourced from Servicebio, China). Simultaneously, HepG2 cells were exposed to primary antibodies against PPARα and NFκB p65 (both obtained from Proteintech, China). For the cellular experiments, to evaluate the levels of reactive oxygen species (ROS), HepG2 cells were incubated with 2′,7′-dichlorofluorescin diacetate (DCFDA) (Beyotime, China) for 30 min, in accordance with the manufacturer’s protocol. The samples were then treated with the appropriate secondary antibodies. To observe lipid droplets, HepG2 cells were stained with BODIPY 493/503 (Thermo Fisher Scientific) for a duration of 30 min. All images were acquired using a digital imaging scanner (KONFOONG BIOINFORMATION TECH CO., LTD, Ningbo, China).

### 2.10 Reactive oxygen species (ROS) analysis

To evaluate intracellular reactive oxygen species (ROS) levels, cells were cultured in 12-well plates and subjected to the indicated treatments. Subsequently, cells were incubated with 5 μM dihydroethidium (DHE; KeyGEN Biotech, Jiangsu, China) for 30 min at 37 C in light-protected conditions. After incubation, cells were rinsed thrice with phosphate-buffered saline (PBS) and examined under a fluorescence microscope (Nikon, magnification ×400). For *in vivo* ROS detection, mice received a tail vein injection of DHE (10 mM, 10 μL) 40 min prior to euthanasia. Hepatic tissues were then collected, and ROS fluorescence intensity was quantified using the LB983 NC100 system (Berthold Technologies) at the Science and Technology Innovation Center, Guangzhou University of Chinese Medicine.

### 2.11 RNA-sequencing analysis

RNA sequencing was performed and subsequently analyzed by Berry Genomics Corporation (Beijing, China), following the methodology outlined in previous studies. In brief, 1 µg of RNA extracted from the specified samples served as the input material for RNA library preparation. Sequencing was performed utilizing the NEBNext^®^ Ultra™ RNA Library Prep Kit for Illumina^®^ (NEB, USA) in accordance with the manufacturer’s instructions, with an Index code added to each sample to enable sequence attribution. The clustering of index-labeled samples was performed using a cBot Cluster Generation System and TruSeq PE Cluster Kit v3-cBot-HS (Illumina), in accordance with the manufacturer’s instructions. The prepared libraries were sequenced on an Illumina NovaSeq platform, producing 150 bp paired-end reads. For the subsequent data analysis, HTSeq version 0.6.1 was utilized.

### 2.12 Western blot (WB) analysis

Protein extracts obtained from the designated cells or liver tissues were subjected to lysis and homogenization using RIPA lysis buffer, and the protein concentrations were quantified using a bicinchoninic acid (BCA) protein assay kit (Beyotime Biotechnology, Shanghai, China). Subsequently, 80–100 μg of the protein samples were loaded onto a 10% SDS-PAGE gel, and the proteins were transferred onto polyvinylidene fluoride (PVDF) membranes. Western blotting was performed with specific primary antibodies, including anti-AKT, anti-p-AKT, anti-PI3K, anti-p-PI3K, anti-Nrf2, anti-HO-1, and anti-PPARα from Proteintech (Wuhan, China), along with anti-β-actin from Abclonal (Wuhan, China). The resulting images were captured and analyzed using Image Lab v4.0 software.

### 2.13 Quantitative polymerase chain reaction (qPCR)

Total RNA was isolated from the designated cells or liver tissue samples using a Trizol reagent kit (Invitrogen). Subsequently, complementary DNA (cDNA) was synthesized using a high-efficiency cDNA reverse transcription kit. Quantitative PCR (q-PCR) was then performed on the resulting cDNA using PowerUp™ SYBR™ Green Master Mix, with β-actin serving as the reference gene for normalization. The specific primer sequences are listed in Supplementary Material 3.

### 2.14 Statistical analysis

The data were processed and analyzed using GraphPad Prism 9.0, with normality and homogeneity of variance tested for each dataset. For data that followed a normal distribution, one-way ANOVA was utilized for intergroup comparisons, whereas the nonparametric rank-sum test was applied to datasets that deviated from normality. A P-value of less than 0.05 was deemed statistically significant, indicating a substantial difference between the groups.

## 3 Results

### 3.1 Network Pharmacology Prediction of JPD on MASH

**FIGURE 1:**
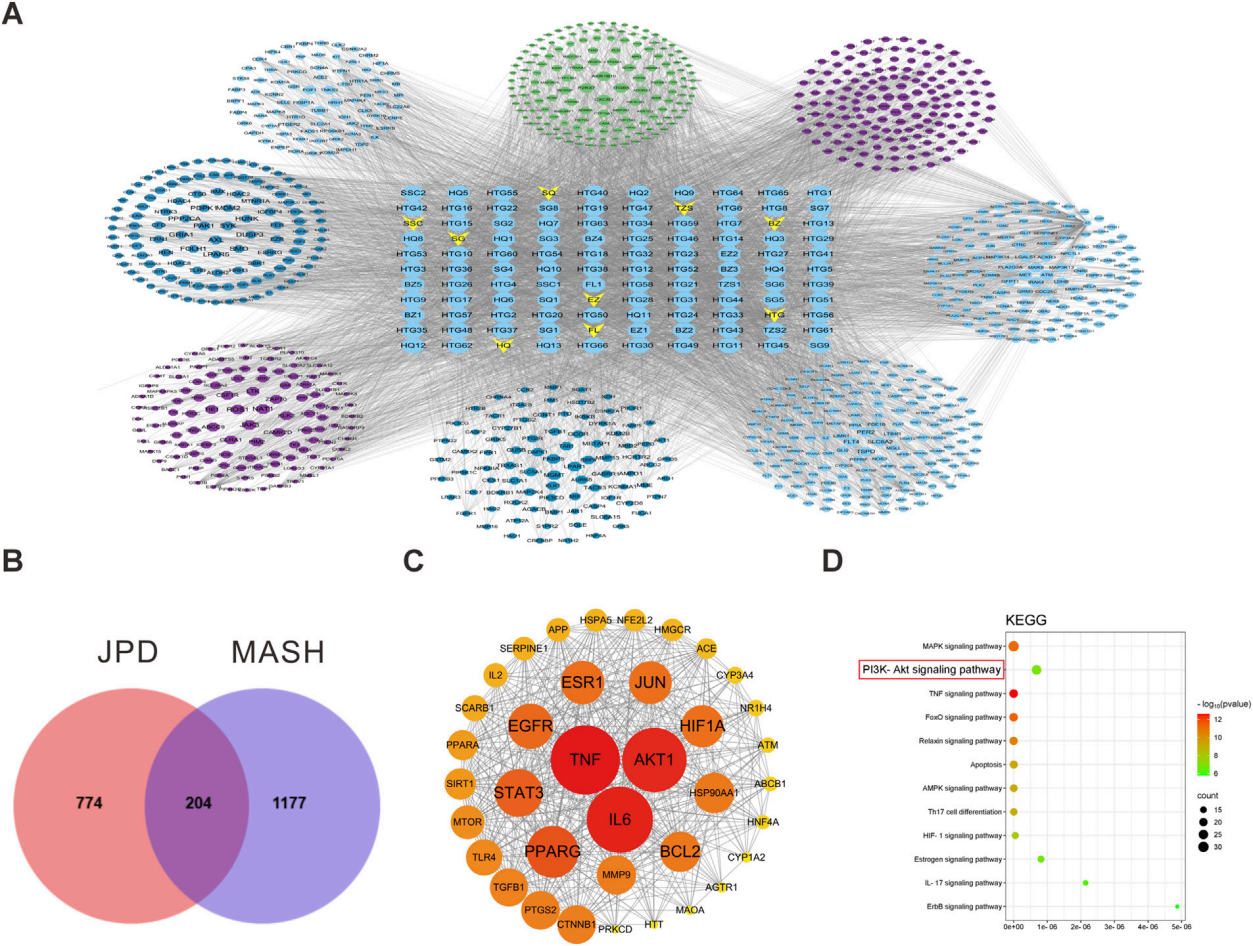
Network Pharmacology Prediction of JPD on MASH. **(A)** The high quality ingredients and corresponding targets of JPD corresponding to the target network of MASH; **(B)** Intersection of JPD and MASH Targets; **(C)** Visualization network diagram of core targets (the color intensity reflects the degree of critical importance of the gene, with darker shades indicating higher significance levels); **(D)** plot of KEGG pathway terms analysis.

To elucidate the potential bioactive compounds of JPD from the TCMSP database, two classical ADME-related parameters, namely, oral bioavailability (OB) and drug-likeness (DL), were employed as screening criteria. As illustrated in Figure 1A, a total of 101 pharmacologically active constituents were identified within the formulation. By intersecting the predicted targets of JPD with MASH-associated targets, 204 shared targets were discovered (Figure 1B). Utilizing Cytoscape 3.7.0 for network topology analysis, the top three hub targets with the highest degree values were determined, including TNF (degree = 135), IL6 (degree = 130), and AKT1 (degree = 127) (Figure 1C). To further validate the functional relevance of these target proteins, KEGG pathway enrichment analysis was performed on the 204 common genes, revealing the top 10 significantly enriched signaling pathways (Figure 1D). Notably, inflammation-associated pathways, such as the MAPK signaling pathway (hsa04010), the PI3K-AKT signaling pathway (hsa04151), and the TNF signaling pathway (hsa04668), were identified as pivotal mechanisms underlying the therapeutic effects of JPD against MASH. These findings suggest that the anti-inflammatory regulatory effect of JPD may serve as a crucial mechanism in its intervention against MASH.

### 3.2 Interactions between active ingredients and targets

Based on the network pharmacology and UHPLC-QTOF-MS analysis results, we prioritized the eight most abundant and bioactive compounds in JPD, specific data are provided in Supplementary Material 4. Molecular docking simulations were subsequently employed to model the binding interactions, aiming to further evaluate the potential interaction patterns between the key constituents of JPD and their corresponding core target proteins, specifically AKT1, NRF2 and PPARα. This approach aimed to provide insights into the potential drug-target binding mechanisms.

**FIGURE 2:**
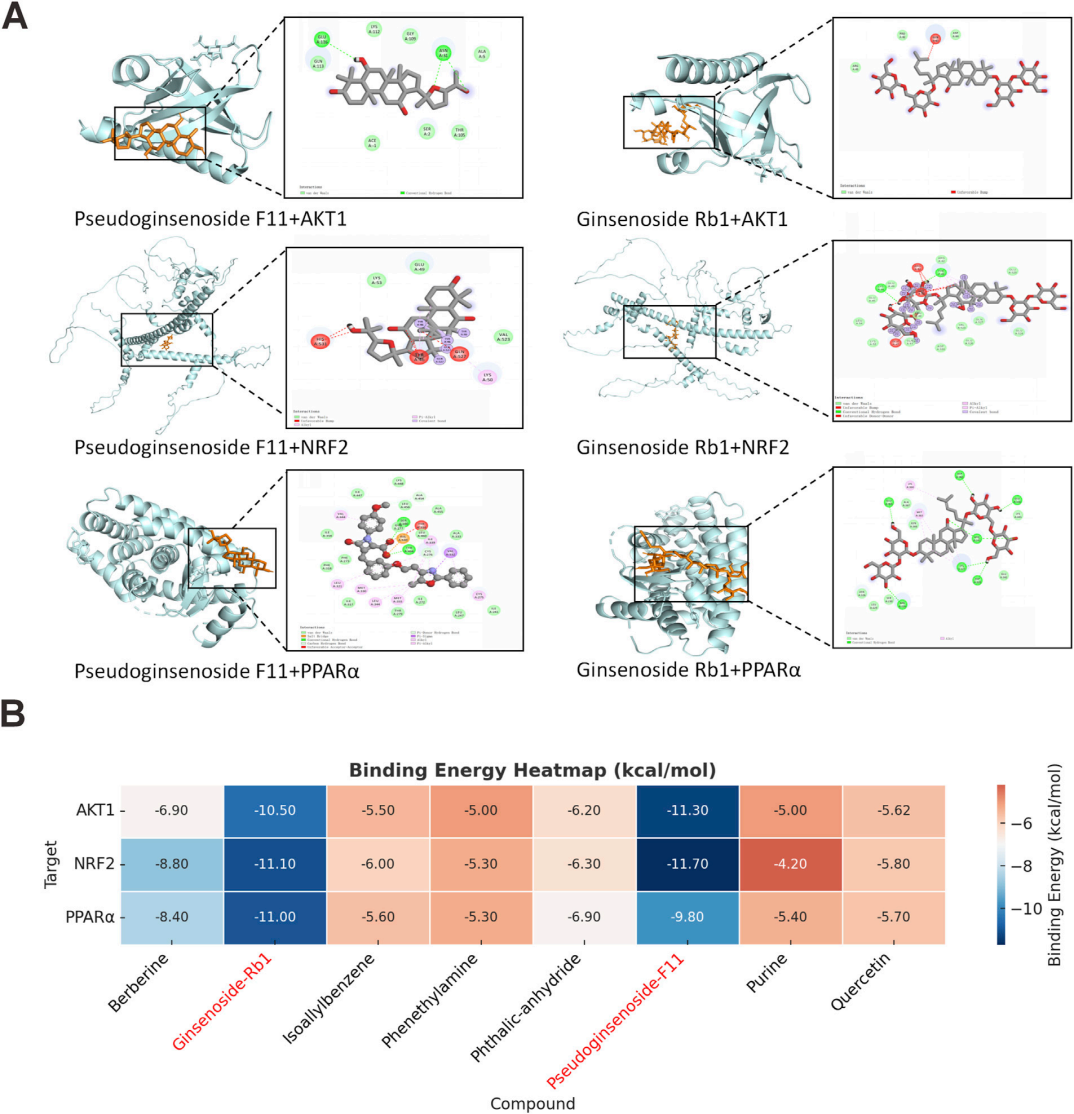
Molecular docking analysis revealed the bond pattern of JPD-components and MASH-targets. **(A)** the bond pattern of AKT1, NRF2, PPARα and Pseudoginsenoside F11, and Ginsenoside Rb1; **(B)** Heatmap visualization of molecular docking binding energies for bioactive compounds (Quercetin, Berberine, Pseudoginsenoside F11, Ginsenoside Rb1, Isoallylbenzene, Phthalic anhydride, Purine, and Phenethylamine) with key therapeutic targets (AKT1, NRF2, and PPARα).

Molecular docking analysis revealed strong binding interactions between the bioactive compounds and key therapeutic targets, with ginsenoside Rb1 exhibiting particularly high affinity for NRF2 (ΔG = −11.10 kcal/mol) and PPARα (ΔG = −11.00 kcal/mol), while Pseudoginsenoside F11 showed robust binding to AKT1 (ΔG = −11.30 kcal/mol). All observed binding energies (≤-4.2 kcal/mol) indicated spontaneous and stable molecular interactions, suggesting these compounds may significantly modulate the respective signaling pathways. The molecular docking results indicated that the primary active constituents of JPD exhibited strong binding affinities with AKT1, PPARα, and NRF2 (Figures 2A,B). These findings align with prior network pharmacology analyses, further substantiating the potential of JPD in targeting these key proteins involved in disease modulation.

### 3.3 JPD effectively mitigated LPS-induced inflammatory responses and suppressed lipid accumulation in HepG2 cells

**FIGURE 3:**
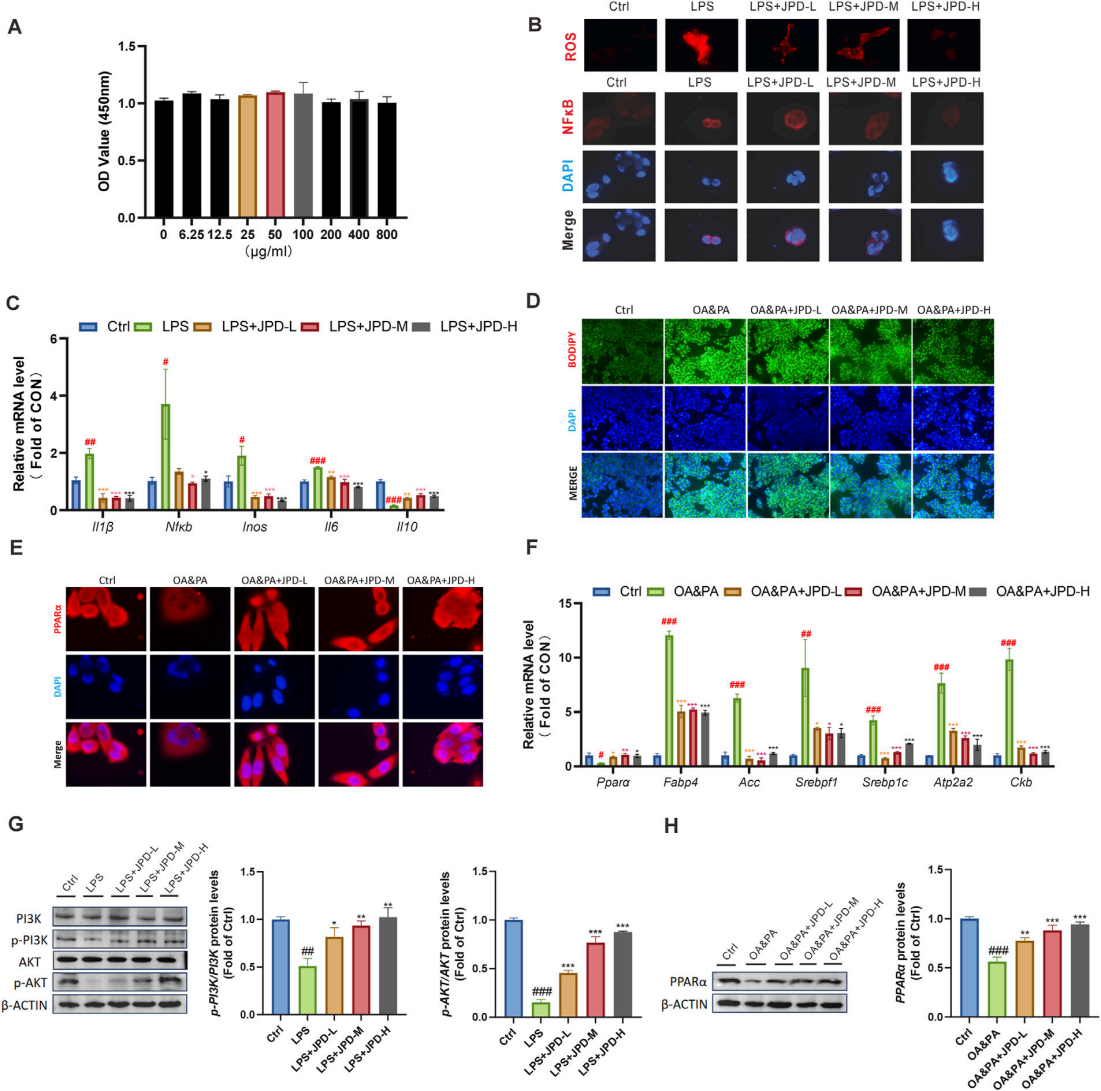
JPD blocked LPS-induced inflammatory responses and lipid accumulation in HepG2 Cells. **(A)** CCK8 assay; **(B)** ROS level and NF-κB expression of cells (100X); **(C)** mRNA levels of inflammatiom-related genes; **(D)** BODIPY-stained cells (40X); **(E)** PPARα expression of cells (100X); **(F)** mRNA levels of lipid metabolism-related genes. **(G,H)** WB analysis of AKT, p-AKT, PI3K, p-PI3K and PPARα protein levels. All data are presented as the mean ± SEM (n = 3). ^#^ *P* < 0.05; ^##^ *P* < 0.01; ^###^ *P* < 0.001 vs Ctrl group; *P < 0.05; **P < 0.01; ***P < 0.001 vs OA&PA group.

JPD was predicted to possess regulatory potential over inflammatory responses and lipid accumulation based on the aforementioned findings. To validate this hypothesis, its impact on inflammation was assessed in LPS-stimulated HepG2 cells. As illustrated in Figure 3A, results from the CCK-8 assay demonstrated that varying concentrations of JPD exhibited minimal cytotoxicity toward HepG2 cells. LPS exposure promoted intracellular ROS production and nuclear NF-κB expression (Figure 3B), whereas pretreatment with JPD significantly attenuated LPS-induced cytokine secretion and substantially downregulated the mRNA expression levels of Il1b, Inos, Il6, and Nfkb, while concurrently upregulating Il10 expression (Figure 3C). Subsequently, the anti-steatosis effects of JPD on hepatocytes were further explored using an OA&PA-induced *in vitro* steatosis model in HepG2 cells. JPD treatment significantly reduced the number and size of lipid droplets accumulated due to OA&PA stimulation (Figure 3D). Additionally, qPCR analysis was performed to evaluate the expression levels of genes associated with lipid synthesis and metabolism. As shown in Figures 3E,F, JPD treatment led to an upregulation of both mRNA and protein expression levels of PPARα in HepG2 cells. Moreover, the mRNA levels of Acc and its upstream regulator Srebp1 were significantly decreased following JPD treatment compared with the control and OA&PA groups. Furthermore, JPD administration suppressed the expression of genes related to lipid synthesis and thermogenesis, including Fabp4, Atp2a2, and Ckb (Figure 3F). Western blot analysis was performed to evaluate the protein expression levels of p-PI3K (T458), p-AKT (S473), and PPARα in HepG2 cells treated with LPS and OA/PA following JPD treatment (Figures 3G,H). Quantitative analysis demonstrated significant upregulation of PPARα expression and enhanced phosphorylation of PI3K (T458) and AKT (S473), with the most robust activation observed in the high-dose JPD treatment group Collectively, these findings suggest that JPD effectively mitigates LPS-induced inflammatory responses and oxidative stress while ameliorating OA&PA-triggered lipid accumulation in HepG2 cells.

### 3.4 JPD alleviated liver injury in the liver of MCD mice

**FIGURE 4:**
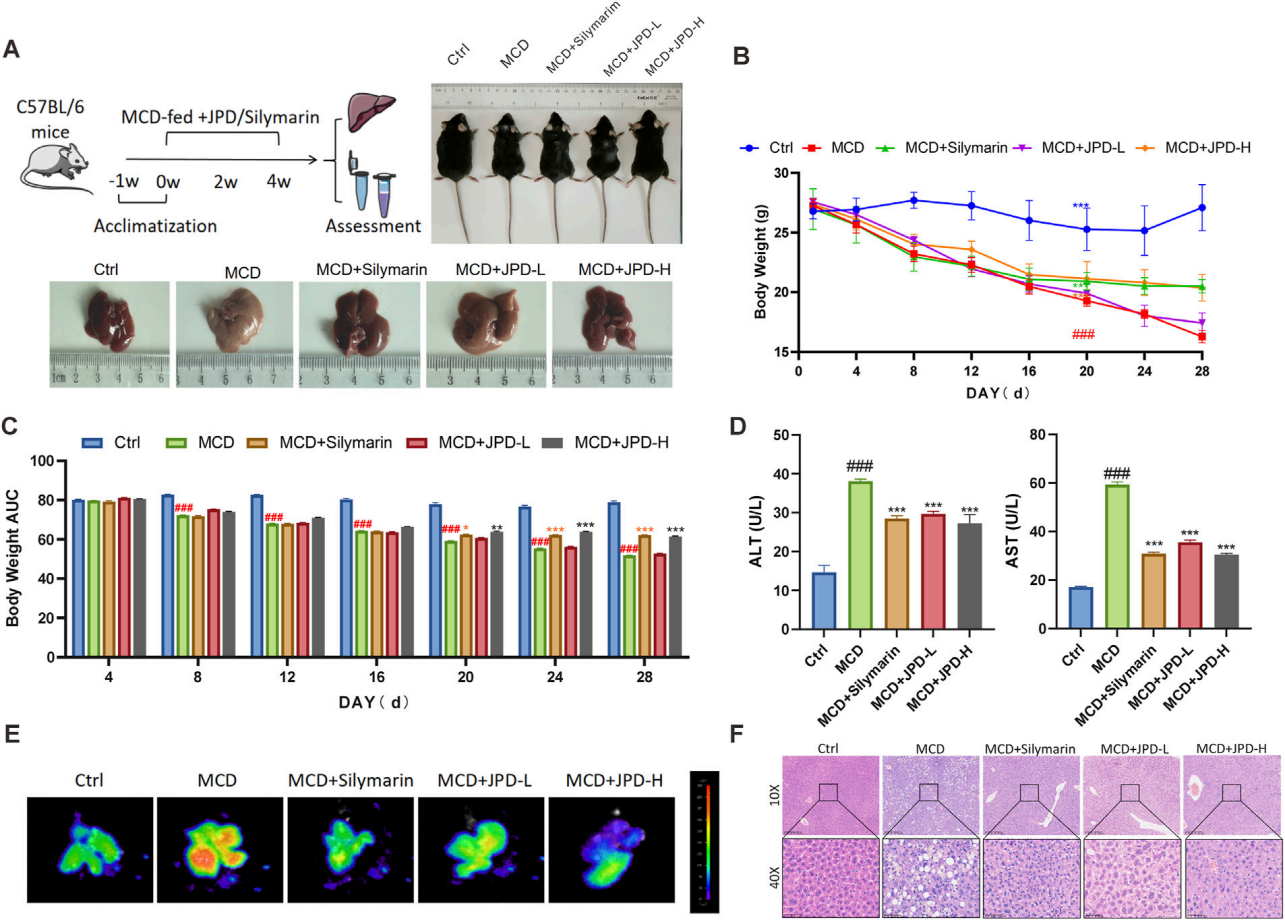
JPD alleviated liver injury in the liver of MCD mice. **(A)** *In vivo* experimental procedure and representative images of mice and their livers; **(B,C)** Body weight change of mice in each group and AUC (n = 6); **(D)** serum ALT and AST levels; **(E)** The liver ROS levels; **(F)** representative images of H&E staining of the liver tissue sections. All data are presented as the mean ± SEM (n = 6). ^#^ *P* < 0.05; ^##^ *P* < 0.01; ^###^ *P* < 0.001 vs Ctrl group; *P < 0.05; **P < 0.01; ***P < 0.001 vs MCD group.

To investigate the potential therapeutic effect of JPD in treating MASH, MCD-fed mice were gavaged with JPD or Silymarin for 4 weeks and the serum and livers of mice were collected for further detection (Figure 4A). JPD and Silymarin treatment significantly delayed weight loss caused by MCD from the 20th day (Figures 4B,C). Additionally, biochemical analyses showed a dose-dependent decrease in serum AST and ALT levels with the JPD treatment (Figure 4D). Concurrently, JPD also reduced the hepatic ROS production, inflammation and lipid deposition induced by MCD (Figures 4E,F). Overall, JPD exerted a pronounced hepatoprotective effect against MCD-induced liver injury in mice.

### 3.5 JPD alleviated inflammation in the hepatic tissue of MCD diet-induced mice

**FIGURE 5:**
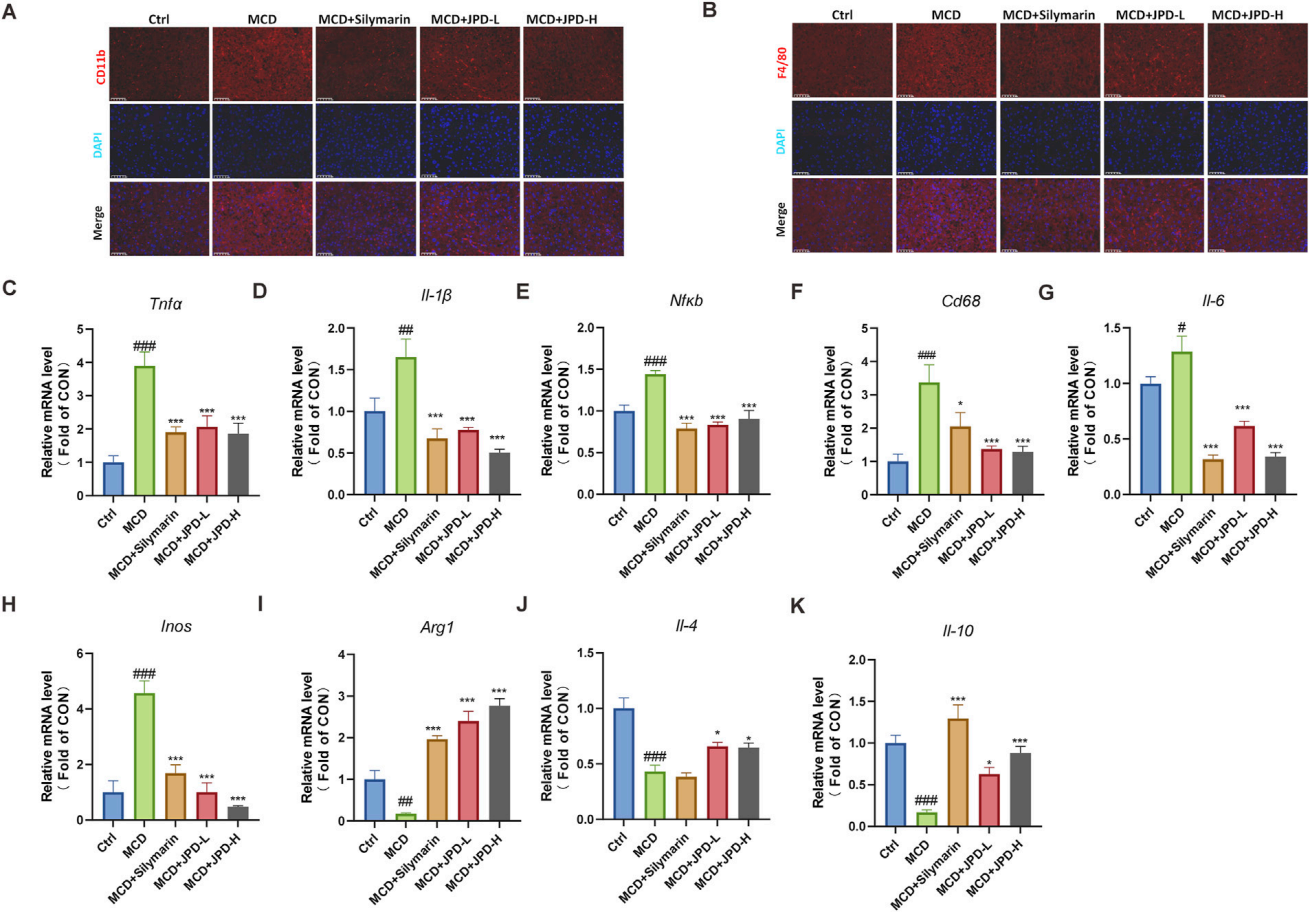
JPD ameliorated hepatic inflammatory response in the liver of MCD mice. **(A,B)** Immunofluorescence analysis of CD11b and F4/80 (50 μm); **(C–H)** mRNA levels of pro-inflammation-related genes; **(I–K)** mRNA levels of anti-inflammation-related genes. All data are presented as the mean ± SEM (n = 6). ^#^ *P* < 0.05; ^##^ *P* < 0.01; ^###^ *P* < 0.001 vs Ctrl group; *P < 0.05; **P < 0.01; ***P < 0.001 vs MCD group.

To further explore the protective effect of JPD, the inflammatory level was detected. As shown in Figures 5A,B, the macrophage markers including F4/80 and CD11b in the livers of MCD mice exhibited a significant increase, whereas treatment with JPD and Silymarin resulted in a reduction of fluorescence intensity for these marker. Meanwhile, JPD treatment downregulated the mRNA levels of pro-inflammatory factor in the liver (Figures 5C–H), and also upregulated the mRNA levels of anti-inflammatory factor in the liver (Figures 5I–K). The PI3K/Akt signaling pathway, which played a critical role in iInflammatory response, was predicted as the potential mechanism involved in JPD treatment of MASH through network pharmacology analysis as mentioned in Figure 1D. These results indicated that JPD effectively reduced inflammation in the liver.

### 3.6 JPD reduced oxidative stress in MCD diet-induced mice

**FIGURE 6:**
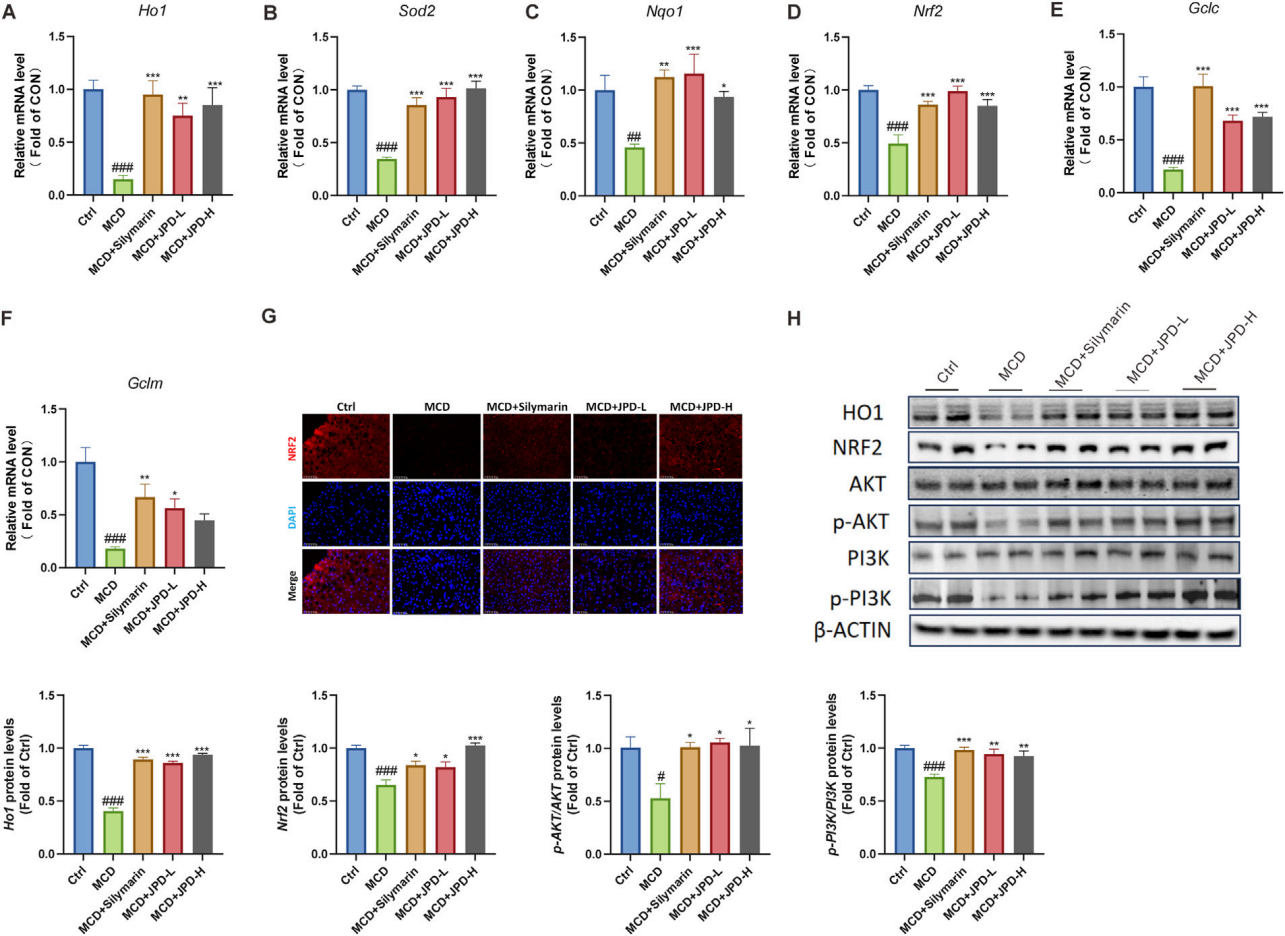
JPD reduced oxidative stress in the liver of MCD mice. **(A–F)** mRNA levels of anti-oxidative stress related genes; **(G)** NRF2 expression of the liver; **(H)** WB analysis of HO1, NRF2, AKT, p-AKT, PI3K and p-PI3K protein levels and Gray Value Analysis of Western blot. All data are presented as the mean ± SEM (n = 6). ^#^ *P* < 0.05; ^##^ *P* < 0.01; ^###^ *P* < 0.001 vs Ctrl group; *P < 0.05; **P < 0.01; ***P < 0.001 vs MCD group.

To further explore the protective effect of JPD, the oxidative stress level was detected. As shown in Figures 6A–F, JPD treatment upregulated the mRNA levels of anti-oxidant genes, including Ho1, Sod2, Nqo1, Nrf2, Gclc, Gclm. Further evidence was found in the result of immunofluorescence, which showed that MCD significantly improved Nrf2 expression (Figure 6G). Also, the results of WB analysis further confirmed the regulation of JPD on Nrf2/HO1 signaling pathway, which was closed related to oxidative stress. These results indicated that JPD improved oxidative stress in MCD mice. The PI3K/Akt signaling pathway, which played a critical role in oxidative stress response, was predicted as the potential mechanism involved in JPD treatment of MASH through network pharmacology analysis as mentioned in Figure 1D. It was found that the JPD treatment could significantly elevated the PI3K/Akt signaling pathway by elevating the phosphorylated protein levels of PI3K and Akt (Figure 6H). These results confirmed that JPD activated the PI3K/AKT-Nrf2-HO1 signalling pathway and suppressed the MCD-induced oxidative stress in the liver.

### 3.7 JPD reduced lipid accumulation in MCD diet-induced mice

**FIGURE 7:**
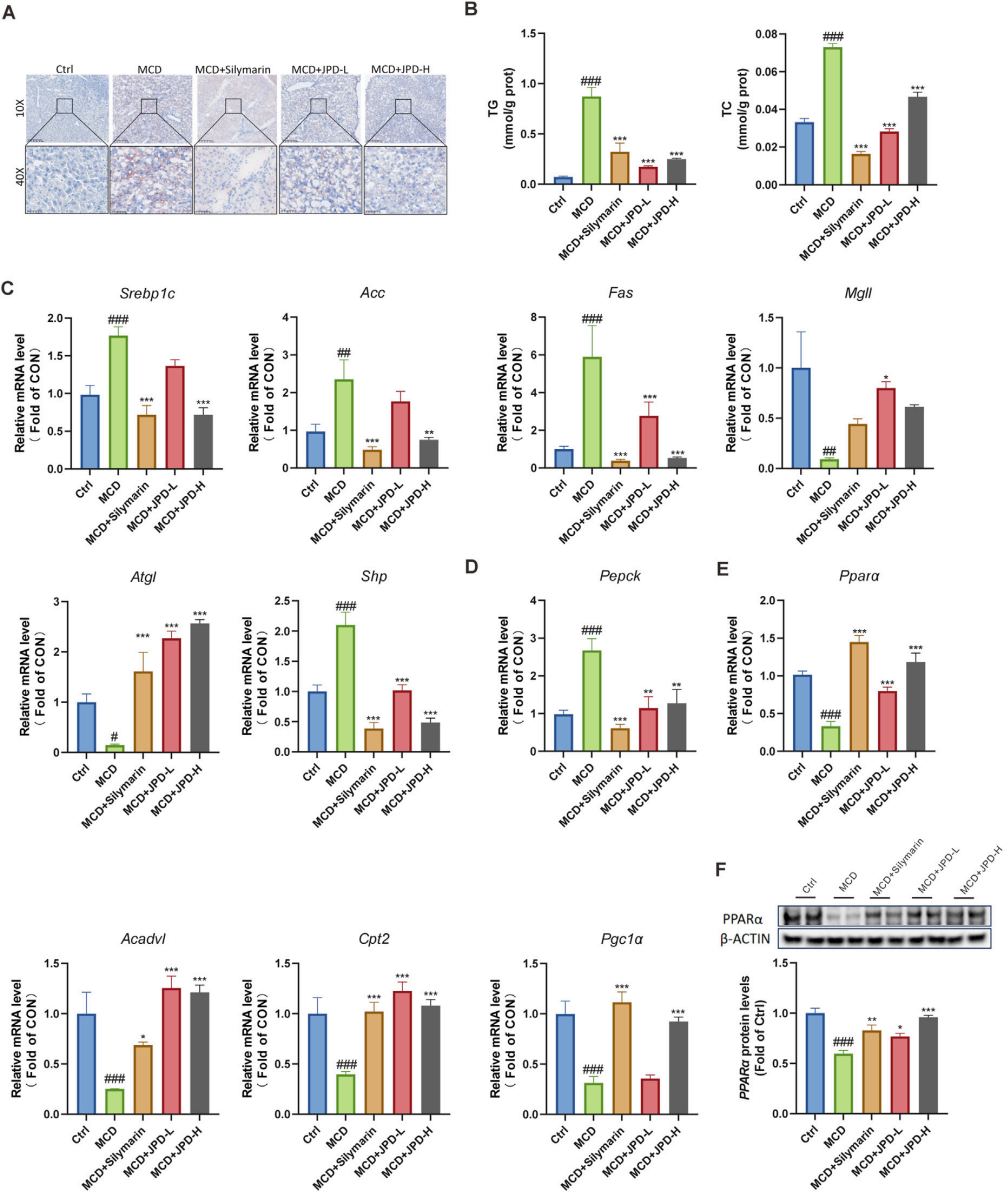
JPD reduced lipid accumulation in the liver of MCD mice. JPD improves MCD diet-induced MASH by reducing lipid accumulation. **(A)** Oil Red O staining of liver tissue sections; **(B)** Serum TG and TC levels; **(C–E)** mRNA levels of lipolytic genes (*Srebp1c, Acc, Fas, Mgll, Atgl, Shp*), glycolytic gene (*Pepck*) and FAO-related genes (*Pparα, Acadvl, Cpt2, Pgc1α*) w; **(F)** WB analysis of PPARα protein levels. All data are presented as the mean ± SEM (n = 6). ^#^ *P* < 0.05; ^##^ *P* < 0.01; ^###^ *P* < 0.001 vs Ctrl group; *P < 0.05; **P < 0.01; ***P < 0.001 vs MCD group.

To further explore the protective effect of JPD, the lipid accumulation level was detected. The results from Oil red O staining showed the intracellular steatosis in the liver in MCD group, as compared with JPD group, whereas the histological changes induced by MCD diet were restored by JPD treatment (Figure 7A). Moreover, JPD effectively reduces the levels of TG and TC in the liver of MCD-induced mice (Figure 7B). qPCR analysis was also evident that JPD treatment group, compared to the MCD group, its mRNA levels of lipolytic genes (Srebp1c, Acc, Fas, Mgll, Atgl, Shp) and glycolytic gene (Pepck) were decreased, while the mRNA levels of FAO-related genes (Pparα, Acadvl, Cpt2, Pgc1α) were upregulated (Figures 7C–E). As shown in Figure 7F, treatment with JPD resulted in an increase in the protein expression of PPARα in the liver of MCD mice, in contrast to the control group. These results suggest that JPD prevents the progression of MASH by promoting fatty acid oxidation pathways, thereby reducing lipid accumulation. Accumulating evidence underscores the pivotal role of impaired fatty acid oxidation (FAO) in MASH progression (Syed-Abdul, 2023a). Our results demonstrate that JPD treatment upregulates PPARα expression (p < 0.05), which may attenuate hepatic lipotoxicity through coordinated activation of mitochondrial β-oxidation (via CPT2 induction)and peroxisomal FAO, thereby reducing lipid accumulation and oxidative stress (Kersten and Stienstra, 2017a). Suggesting JPD’s multi-target potential in interrupting the MASH pathogenic cascade.

### 3.8 JPD ameliorated MCD-induced MASH by regulating PPAR signaling pathway and PI3K/AKT signaling pathway

**FIGURE 8:**
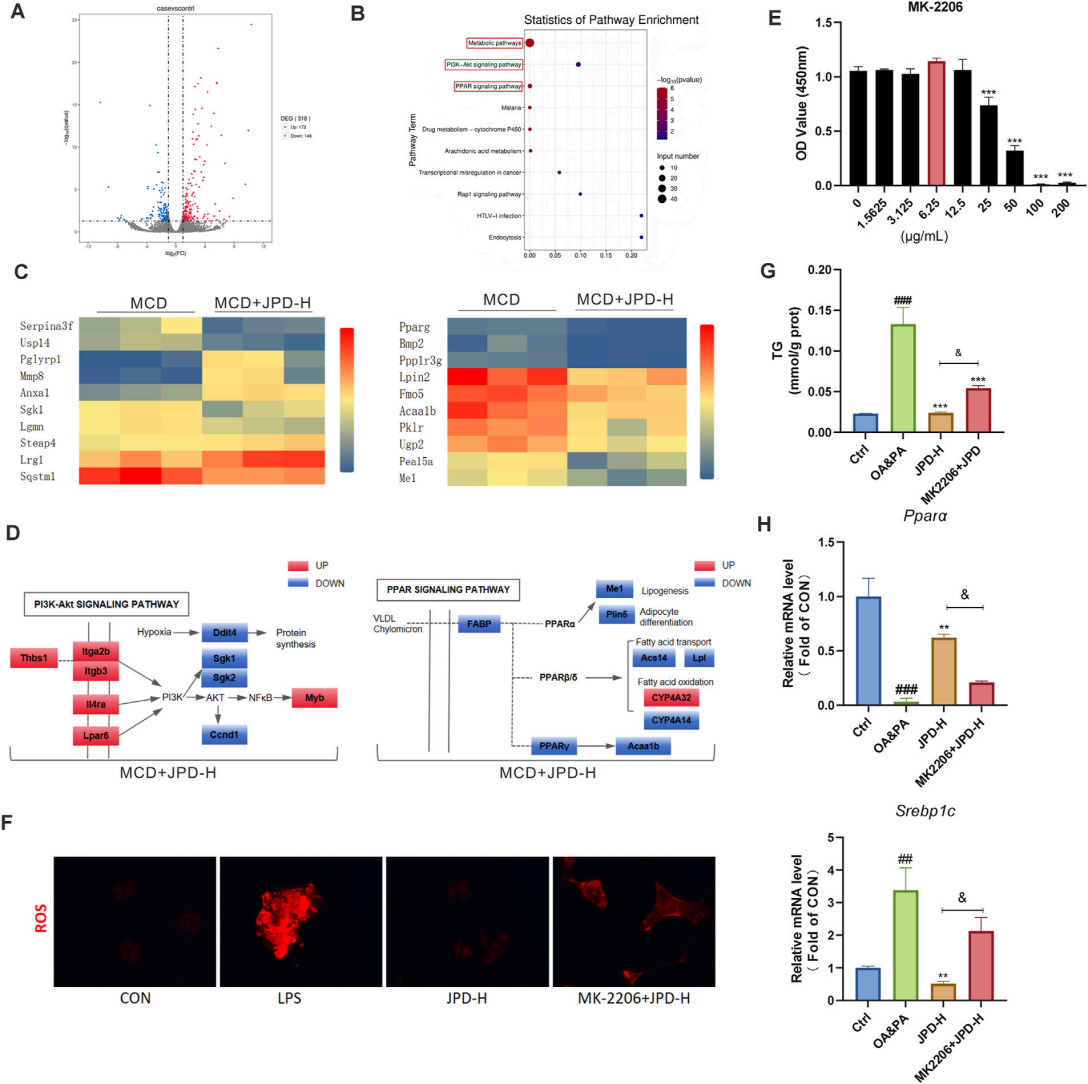
JPD ameliorated MCD-induced MASH by regulating PPAR signaling pathway and PI3K/AKT signaling pathway. **(A)** Volcano plots; **(B)** Statistics of pathway enrichment; **(C,D)** heatmaps and Gene pathway maps of PPAR signaling pathway- and PI3K-Akt signaling pathway-related genes. **(E)** The dose-dependent effect of AKT1 inhibitor (MK-2206 2HCL)on the cell viability of HepG2 cells; **(F)** ROS expression in HepG2 cells; **(G)** TG level in OA&PA-induced cells; **(H)** mRNA levels of *Ppara*, and *Srebp1c*. All data are presented as the mean ± SEM (n = 3). #P < 0.05; ##P < 0.01; ###P < 0.001 vs Ctrl group; *P < 0.05; **P < 0.01; ***P < 0.001 vs OA&PA group. *and < 0.05; **and < 0.01; ***and < 0.001 vs. high-dose treatment group (MCD + JPD-H).

To verify the mechanism predicted by network pharmacology, transcriptomic sequencing analysis was performed on the livers of MCD mice. The results showed a volcano plot enrichment of 318 genes, with 172 genes being upregulated and 146 genes downregulated (Figure 8A). KEGG pathway analysis revealed enrichment in the PPAR signaling pathway, metabolic pathways as well as PI3K-Akt signaling pathway (Figure 8B). As shown in Figures 8C,D, PPAR signaling pathway- and PI3K-Akt signaling pathway-related genes were regulated by JPD. To further verify the mechanism of JPD, the AKT1 inhibitor were used in LPS- and OA&PA-induced HepG2 cells. In our study, the results of the CCK-8 assay show that specific Akt inhibitor (MK-2206 2HCL, 6.25 μg/mL) was not toxic to HepG2 cells (Figure 8E). As shown in Figures 8F–H, the AKT1 inhibitor significantly reduced the anti-ROS generation anti-lipid accumulation effect of JPD. Furthermore, they reduced the role of JPD in PPARα pathway (Figure 8H). The results indicated that the JPD treatment regulated the PPAR signaling pathway and PI3K/AKT signaling pathway.

## 4 Discussion

MASH, as a common chronic liver disease, has a complex pathogenesis involving inflammation, lipid metabolism disorders, oxidative stress, and other multiple links, posing a serious threat to human health (Xu et al., 2022b). The traditional Chinese medicinal formulation JPD has demonstrated promising therapeutic benefits in the treatment of liver diseases in clinical settings; however, its precise mechanisms of action remain poorly understood. This investigation integrates network pharmacology with experimental approaches to provide an in-depth analysis of the underlying mechanisms through which JPD exerts its effects in the management of MASH.

The MCD diet-induced model of MASH is widely recognized for its reproducibility and reliability. Mice subjected to an MCD diet for 4–8 weeks develop liver conditions including steatosis, inflammation, and even fibrosis, closely resembling the pathophysiological characteristics of human MASH (Dong et al., 2024; Duan et al., 2017; Zhao et al., 2020b). Silymarin, commonly used as a positive control *in vivo* studies, has been shown to effectively treat MASH in randomized clinical trials (Wah et al., 2017), and is widely regarded as a reliable therapeutic agent in the management of liver diseases (Abenavoli et al., 2018; Xiang et al., 2016). In the present investigation, we observed that JPD alleviated several critical factors contributing to MASH, such as steatosis, inflammation, and oxidative stress. Moreover, JPD demonstrated protective effects by halting the progression of MASH through the inhibition of these key pathological processes, exhibiting effects comparable to those of silymarin. Therefore, additional studies are warranted to thoroughly investigate the underlying mechanisms by which JPD mitigates MASH.

The PI3K/AKT pathway assumes a pivotal role in the activation of inflammatory cells and the release of inflammatory mediators, emerging as a critical nexus in the progression of MASH or even hepatocellular carcinoma (HCC) (Becattini et al., 2021). When the liver sustains damage, inflammatory cells such as macrophages are recruited into the hepatic tissue, and the activated PI3K/AKT-NRF2 pathway can regulate macrophages to release inflammatory factors like TNF-α and IL-6 (Hu et al., 2021). Simultaneously, under MASH conditions, within liver cells, The PI3K/AKT signaling pathway regulates IκB phosphorylation, thereby modulating nuclear translocation of NF-κB and subsequent inflammatory responses (Li et al., 2021). These inflammatory responses constitute a pivotal driver of MASH progression (Zhang et al., 2019). The findings presented in this study illustrate that JPD substantially mitigated the pathological manifestations of MASH, as evidenced by a notable reduction in AST/ALT levels, amelioration of hepatic steatosis, diminished oxidative stress, and decreased inflammatory infiltration. Mechanistically, JPD activates the PI3K/AKT pathway, markedly increases the expression of the oxidative stress marker NRF2,Additionally, JPD significantly reducing the transcriptional and protein levels of TNFα, IL1β, and NFκB, indicating that its therapeutic effect on MASH is primarily due to enhanced antioxidant capacity,further suppress the expression of inflammatory genes. These findings suggest that JPD mitigates hepatic inflammation and oxidative stress through the activating PI3K/AKT pathway,enhancing antioxidant capacity and reducing the expressions of downstream inflammatory targets such as TNFα and NFκB, thereby inhibiting the progression of liver inflammation.

In the coming years, non-alcoholic steatohepatitis (MASH) is expected to surpass hepatitis C virus infection as the leading cause of liver transplants globally (Paik et al., 2020; Terrault et al., 2023). Consequently, the need for identifying effective therapeutic targets has become more pressing than ever. The drug development landscape for MASH can be categorized into five primary approaches: lipid modulators, such as PPARα/γ agonists; hypoglycemic agents, including SGLT1/2 inhibitors; bile acid-related compounds, like FXR agonists; anti-inflammatory drugs, such as ASK-1 inhibitors; and anti-fibrotic therapies (Huang et al., 2021; Shen and Lu, 2021a; Friedman et al., 2018). However, despite the promising potential of the FXR agonist obeticholic acid (OCA) in phase III clinical trials, the FDA rejected its approval in June 2020 (Abdelmalek, 2021; Shen and Lu, 2021b). These findings imply that interventions focused on a solitary target might prove inadequate for effectively improving MASH. Instead, treatment strategies may need to be multi-pronged, targeting numerous cellular and molecular processes implicated in the advancement of MASH to achieve favorable outcomes (Chen et al., 2021). PPARα can reduce *de novo* synthesis of fatty acids and maintain the balance of liver lipid metabolism by inhibiting the expression of FAS, ACC, and increasing FAO, which was proved by previous study (Pawlak et al., 2015). As in our study, experimental results indicate that JPD significantly reduces hepatic TG and TC levels in these mice. Not surprisingly, JPD significantly alleviated the hepatic lipid accumulation through regulating lipid metabolism-related genes including Srebp1c, Acc, Fas, et al. Our results demonstrate that JPD attenuates MASH progression by enhancing fatty acid oxidation (FAO) pathways, effectively reducing hepatic lipid accumulation. Consistent with established evidence implicating impaired FAO in MASH pathogenesis (Syed-Abdul, 2023b), our data demonstrate that JPD treatment significantly upregulates PPARα expression (p < 0.05), serving as a central regulator of both mitochondrial β-oxidation (via CPT2 induction) and peroxisomal FAO. This dual activation mitigates lipotoxicity by decreasing lipid deposition and oxidative stress (Kersten and Stienstra, 2017b), highlighting JPD’s multi-target therapeutic potential against key drivers of MASH progression.

Consistent with previous studies, our analysis identified several bioactive constituents in JPD, including the flavonoid quercetin, the alkaloid berberine, and the ginsenoside Rb1, all of which have been demonstrated to possess various pharmacological effects such as anti-inflammatory and metabolic regulatory activities, and previous reports have suggested their potential activity in improving MASH (Cao et al., 2023). For example, quercetin ameliorated dysregulation of lipid metabolism genes via the PI3K/AKT pathway in a diet-induced mouse model of MASH (Pisonero-Vaquero et al., 2015). In the present investigation, molecular docking techniques were employed to conduct an initial analysis of the potential bioactive components of JPD. Consistent with previous studies, our results further confirm the correlation between quercetin and the AKT pathway (Pisonero-Vaquero et al., 2015). In addition, Berberine exerts hepatoprotective effects by inhibiting lipogenesis and gluconeogenesis while improving insulin sensitivity and lipid profiles (Koperska et al., 2022).Ginsenoside Rb1 demonstrates significant improvements in dyslipidemia (Kim et al., 2019). However, the effect of them in treating MASH has not been found. The drawback of this study is the lack of validation of active ingredients of JPD, which is also the direction of our future work.

In this study, the MCD diet was used to induce a MASH mouse model, which is a common approach for investigating the independent mechanisms of hepatocyte lipotoxicity, oxidative stress, and inflammation (Dong et al., 2024; Duan et al., 2017; Zhao et al., 2020a). However, this model has certain limitations. For instance, the MCD model lacks key features of human MASH, such as the typical coexistence of obesity in human patients. In the future, our research will explore alternative modeling methods, such as the high-fat diet (HFD), to provide a more comprehensive understanding of MASH (Tsuchida et al., 2018; Lazarus et al., 2024).

We focused primarily on the analysis of the PI3K/AKT pathway due to its well-established role in the pathogenesis of MASH, particularly in hepatocyte lipotoxicity, oxidative stress, and inflammatory responses in our current study. However, we acknowledge the importance of exploring the crosstalk between the PI3K/AKT and PPARα pathways, as this interaction may significantly influence the progression of MASH. Existing literature has indicated that the PPARα pathway plays a key role in regulating fatty acid oxidation and energy metabolism (Qiu et al., 2023), while the PI3K/AKT pathway is involved in cell survival, metabolic regulation, and inflammatory responses (Ye et al., 2023). Emerging evidence suggests stage-specific therapeutic targets in MASH pathogenesis: PPARα activation demonstrates protective effects against hepatic lipotoxicity during early disease progression by enhancing fatty acid oxidation (Dixon et al., 2025), while subsequent PI3K/AKT pathway activation may promote hepatocyte survival through metabolic reprogramming and anti-apoptotic signaling (Liu et al., 2025). In contrast, during the advanced stages of MASH, overactivation of the PI3K/AKT pathway may exacerbate inflammatory responses, while the activity of PPARα may be suppressed, thereby affecting fatty acid oxidation and energy metabolism (Tu et al., 2025). Emerging evidence reveals complex crosstalk between these pathways, including reciprocal phosphorylation events, shared downstream effectors, and competitive transcription factor binding, as demonstrated in recent systems biology studies. For instance, the PI3K/AKT pathway may regulate the transcriptional activity of PPARα or the expression of its downstream target genes, thereby influencing fatty acid metabolism. Conversely, activation of PPARα may indirectly affect the activity of the PI3K/AKT pathway by modulating the intracellular metabolic state (Iershov et al., 2019). This crosstalk is likely to play a critical role in the progression of MASH, particularly in modulating inflammatory responses and lipid metabolism.

A comprehensive investigation of the interaction between the PI3K/AKT and PPARα pathways is essential for a holistic understanding of MASH pathogenesis. In our future research, we plan to undertake the following steps:first,we will conduct a joint analysis of the PI3K/AKT and PPARα pathways to elucidate their interaction mechanisms in different stages of MASH. Then,we will utilize both *in vitro* cell models and *in vivo* animal models to study the cross-talk between these pathways and its impact on MASH progression. Finally,we will analyze the expression levels and activity states of these pathways in clinical samples to further validate their roles and interactions in MASH. We will incorporate these considerations into our future research and aim to provide a more comprehensive understanding of the pathogenesis of MASH.

## 5 Conclusion

In this research, our findings indicate that JPD alleviated MASH induced by the MCD diet by reducing hepatic lipid accumulation, oxidative stress, and inflammation, likely through the activation of the PI3K/AKT pathway and the PPAR signaling pathway. Given its hepatoprotective effects and lipid-reducing properties, JPD shows promising potential as a novel and effective therapeutic agent for the treatment of MASH. Moreover, based on our current findings, further investigation is warranted to elucidate the precise mechanisms through which these two pathways exert their effects—whether through direct molecular interactions or via crosstalk remains a key focus for future research.

## Data Availability

The datasets presented in this study can be found in online repositories. The names of the repository/repositories and accession number(s) can be found in the article/Supplementary Material.

## Abbreviations

MASH, metabolic dysfunction-associated steatohepatitis; JPD, Jianpi-Huayu-Jiedu formula; MCD, Methionine- and choline-deficient; OA&PA, Oleic acidandpalmitic acid; LPS, Lipopolysaccharide; ER, Endoplasmic reticulum; ROS, Reactive oxygen species; CMM, Chinese Materia Medica; CCK-8, Cell Counting Kit-8; PDB, Protein Data Bank; DMEM, Dulbecco’s Modified Eagle Medium; H&E, Hematoxylin-eosin staining; BCA, Bicinchoninic acid; DCFDA, 2′,7′-dichlorofluorescin diacetate; qPCR, Quantitative polymerase chain reaction; WB, Western blot analysis; cDNA, Complementary DNA; PVDF, Polyvinylidene fluoride; OB, Oral bioavailability; DL, Drug-likeness; HCC, Hepatocellular carcinoma.

## References

[B1] Abdelmalek M. F. (2021). Nonalcoholic fatty liver disease: another leap forward. Nat. Rev. Gastroenterol. Hepatol. 18 (2), 85–86. 10.1038/s41575-020-00406-0 33420415 PMC7791336

[B2] Abenavoli L. Izzo A. A. Milic N. Cicala C. Santini A. Capasso R. (2018). Milk thistle (silybum marianum): a concise overview on its chemistry, pharmacological, and nutraceutical uses in liver diseases. Phytother. Res. 32 (11), 2202–2213. 10.1002/ptr.6171 30080294

[B3] Arguello G. Balboa E. Arrese M. Zanlungo S. (2015). Recent insights on the role of cholesterol in non-alcoholic fatty liver disease. Biochim. Biophys. Acta 1852 (9), 1765–1778. 10.1016/j.bbadis.2015.05.015 26027904

[B4] Becattini B. Breasson L. Sardi C. Zani F. Solinas G. (2021). PI3Kγ promotes obesity-associated hepatocellular carcinoma by regulating metabolism and inflammation. JHEP Rep. 3 (6), 100359. 10.1016/j.jhepr.2021.100359 34704005 PMC8521290

[B5] Cao P. Wang Y. Zhang C. Sullivan M. A. Chen W. Jing X. (2023). Quercetin ameliorates nonalcoholic fatty liver disease (NAFLD) *via* the promotion of AMPK-Mediated hepatic mitophagy. J. Nutr. Biochem. 120, 109414. 10.1016/j.jnutbio.2023.109414 37423322

[B6] Chen Z. Tian R. She Z. Cai J. Li H. (2020). Role of oxidative stress in the pathogenesis of nonalcoholic fatty liver disease. Free. Radic. Biol. Med. 152, 116–141. 10.1016/j.freeradbiomed.2020.02.025 32156524

[B7] Chen M. Xie Y. Gong S. Wang Y. Yu H. Zhou T. (2021). Traditional Chinese medicine in the treatment of nonalcoholic steatohepatitis. Pharmacol. Res. 172, 105849. 10.1016/j.phrs.2021.15849 34450307

[B8] Chen J. Yang S. Luo H. Fu X. Li W. Li B. (2024). Polysaccharide of Atractylodes macrocephala koidz alleviates NAFLD-induced hepatic inflammation in mice by modulating the TLR4/MyD88/NF-κB pathway. Int. Immunopharmacol. 141, 113014. 10.1016/j.intimp.2024.113014 39191120

[B9] Ding L. Sun W. Balaz M. He A. Klug M. Wieland S. (2021). Peroxisomal beta-oxidation acts as a sensor for intracellular fatty acids and regulates lipolysis. Nat. Metab. 3 (12), 1648–1661. 10.1038/s42255-021-00489-2 34903883 PMC8688145

[B10] Dixon E. D. Claudel T. Nardo A. D. Riva A. Fuchs C. D. Mlitz V. (2025). Inhibition of ATGL alleviates MASH *via* impaired PPARα signalling that favours hydrophilic bile acid composition in mice. J. Hepatol. 82 (4), 658–675. 10.1016/j.jhep.2024.09.037 39357546

[B11] Dong J. Chen L. Ye F. Tang J. Liu B. Lin J. (2024). Mic19 depletion impairs endoplasmic reticulum-mitochondrial contacts and mitochondrial lipid metabolism and triggers liver disease. Nat. Commun. 15 (1), 168. 10.1038/s41467-023-44057-6 38168065 PMC10762189

[B12] Duan X. Meng Q. Wang C. Liu Z. Liu Q. Sun H. (2017). Calycosin attenuates triglyceride accumulation and hepatic fibrosis in murine model of non-alcoholic steatohepatitis *via* activating farnesoid X receptor. Phytomedicine 25, 83–92. 10.1016/j.phymed.2016.12.006 28190475

[B13] Filozof C. Chow S. C. Dimick-Santos L. Chen Y. F. Williams R. N. Goldstein B. J. (2017). Clinical endpoints and adaptive clinical trials in precirrhotic nonalcoholic steatohepatitis: facilitating development approaches for an emerging epidemic. Hepatol. Commun. 1 (7), 577–585. 10.1002/hep4.1079 29404480 PMC5721443

[B14] Friedman S. L. Neuschwander-Tetri B. A. Rinella M. Sanyal A. J. (2018). Mechanisms of NAFLD development and therapeutic strategies. Nat. Med. 24 (7), 908–922. 10.1038/s41591-018-0104-9 29967350 PMC6553468

[B15] Gao H. Jin Z. Bandyopadhyay G. Wang G. Zhang D. Rocha K. (2022). Aberrant iron distribution *via* hepatocyte-stellate cell axis drives liver lipogenesis and fibrosis. Cell Metab. 34 (8), 1201–1213.e5. 10.1016/j.cmet.2022.07.006 35921818 PMC9365100

[B16] Harrison S. A. Allen A. M. Dubourg J. Noureddin M. Alkhouri N. (2023). Challenges and opportunities in NASH drug development. Nat. Med. 29 (3), 562–573. 10.1038/s41591-023-02242-6 36894650

[B17] Hu Q. Zhang W. Wu Z. Tian X. Xiang J. Li L. (2021). Baicalin and the liver-gut system: pharmacological bases explaining its therapeutic effects. Pharmacol. Res. 165, 105444. 10.1016/j.phrs.2021.105444 33493657

[B18] Huang D. W. Sherman B. T. Lempicki R. A. (2009). Systematic and integrative analysis of large gene lists using DAVID bioinformatics resources. Nat. Protoc. 4 (1), 44–57. 10.1038/nprot.2008.211 19131956

[B19] Huang R. Guo F. Li Y. Liang Y. Li G. Fu P. (2021). Activation of AMPK by triptolide alleviates nonalcoholic fatty liver disease by improving hepatic lipid metabolism, inflammation and fibrosis. Phytomedicine 92, 153739. 10.1016/j.phymed.2021.153739 34592488

[B20] Iershov A. Nemazanyy I. Alkhoury C. Girard M. Barth E. Cagnard N. (2019). The class 3 PI3K coordinates autophagy and mitochondrial lipid catabolism by controlling nuclear receptor PPARα. Nat. Commun. 10 (1), 1566. 10.1038/s41467-019-09598-9 30952952 PMC6451001

[B21] Kersten S. Stienstra R. (2017a). The role and regulation of the peroxisome proliferator activated receptor alpha in human liver. Biochimie 136, 75–84. 10.1016/j.biochi.2016.12.019 28077274

[B22] Kersten S. Stienstra R. (2017b). The role and regulation of the peroxisome proliferator activated receptor alpha in human liver. Biochimie 136, 75–84. 10.1016/j.biochi.201.12.019 28077274

[B23] Kim J. C. Jeon J. Y. Yang W. S. Kim C. H. Eom D. W. (2019). Combined amelioration of ginsenoside (Rg1, Rb1, and Rg3)-enriched Korean red ginseng and probiotic lactobacillus on non-alcoholic fatty liver disease. Curr. Pharm. Biotechnol. 20 (3), 222–231. 10.2174/1389201020666190311143554 30854954

[B24] Koperska A. Wesolek A. Moszak M. Szulinska M. (2022). Berberine in non-alcoholic fatty liver Disease-A review. Nutrients 14 (17), 3459. 10.3390/nu14173459 36079717 PMC9459907

[B25] Lazarus J. V. Newsome P. N. Francque S. M. Kanwal F. Terrault N. A. Rinella M. E. (2024). Reply: a multi-society Delphi consensus statement on new fatty liver disease nomenclature. Hepatology 79 (3), E93–E94. 10.1097/HEP.0000000000000696 37983810

[B26] Lee D. H. Park J. S. Lee Y. S. Bae S. H. (2022). PERK prevents hepatic lipotoxicity by activating the p62-ULK1 axis-mediated noncanonical KEAP1-Nrf2 pathway. Redox Biol. 50, 102235. 10.1016/j.redox.2022.102235 35091323 PMC8801383

[B27] Li J. Wang T. Liu P. Yang F. Wang X. Zheng W. (2021). Hesperetin ameliorates hepatic oxidative stress and inflammation *via* the PI3K/AKT-Nrf2-ARE pathway in oleic acid-induced HepG2 cells and a rat model of high-fat diet-induced NAFLD. Food Funct. 12 (9), 3898–3918. 10.1039/d0fo02736g 33977953

[B28] Liao Z. Zhang J. Liu B. Yan T. Xu F. Xiao F. (2019). Polysaccharide from okra (Abelmoschus esculentus (L.) moench) improves antioxidant capacity *via* PI3K/AKT pathways and Nrf2 translocation in a type 2 diabetes model. Molecules 24 (10), 1906. 10.3390/molecules24101906 31108940 PMC6571734

[B29] Liu X. Xie Z. H. Liu C. Y. Zhang Y. (2019). Effect of Chinese herbal monomer Hairy Calycosin on Nonalcoholic Fatty liver rats and its mechanism. Comb. Chem. High. Throughput Screen. 22 (3), 194–200. 10.2174/1386207322666190411112814 30973105

[B30] Liu Q. W. Ying Y. M. Zhou J. X. Zhang W. J. Liu Z. X. Jia B. B. (2022). Human amniotic mesenchymal stem cells-derived IGFBP-3, DKK-3, and DKK-1 attenuate liver fibrosis through inhibiting hepatic stellate cell activation by blocking Wnt/β-catenin signaling pathway in mice. Stem Cell Res. Ther. 13 (1), 224. 10.1186/s13287-022-02906-z 35659360 PMC9166579

[B31] Liu R. Zhang Y. Liu M. Shang Z. Song S. Zhang Y. (2025). Natural molecule isoliquiritigenin mitigates MASH and liver fibrosis in mice by promoting autophagy through the PI3K/Akt signaling pathway. J. Nutr. Biochem. 136, 109808. 10.1016/j.jnutbio.2024.109808 39571827

[B32] Mah C. Y. Nguyen A. Niijima T. Helm M. Dehairs J. Ryan F. J. (2024). Peroxisomal beta-oxidation enzyme, DECR2, regulates lipid metabolism and promotes treatment resistance in advanced prostate cancer. Br. J. Cancer. 130 (5), 741–754. 10.1038/s41416-023-02557-8 38216720 PMC10912652

[B33] Paik J. M. Golabi P. Younossi Y. Mishra A. Younossi Z. M. (2020). Changes in the global burden of chronic liver diseases from 2012 to 2017: the growing impact of NAFLD. Hepatology 72 (5), 1605–1616. 10.1002/hep.31173 32043613

[B34] Pawlak M. Lefebvre P. Staels B. (2015). Molecular mechanism of PPARα action and its impact on lipid metabolism, inflammation and fibrosis in non-alcoholic fatty liver disease. J. Hepatol. 62 (3), 720–733. 10.1016/j.jhep.2014.10.039 25450203

[B35] Pisonero-Vaquero S. Martinez-Ferreras A. Garcia-Mediavilla M. V. Martinez-Florez S. Fernandez A. Benet M. (2015). Quercetin ameliorates dysregulation of lipid metabolism genes *via* the PI3K/AKT pathway in a diet-induced mouse model of nonalcoholic fatty liver disease. Mol. Nutr. Food Res. 59 (5), 879–893. 10.1002/mnfr.201400913 25712622

[B36] Qiu Y. Y. Zhang J. Zeng F. Y. Zhu Y. Z. (2023). Roles of the peroxisome proliferator-activated receptors (PPARs) in the pathogenesis of nonalcoholic fatty liver disease (NAFLD). Pharmacol. Res. 192, 106786. 10.1016/j.phrs.2023.106786 37146924

[B37] Rinella M. E. Lazarus J. V. Ratziu V. Francque S. M. Sanyal A. J. Kanwal F. (2023). A multisociety Delphi consensus statement on new fatty liver disease nomenclature. Hepatology 78 (6), 1966–1986. 10.1097/HEP.0000000000000520 37363821 PMC10653297

[B38] Ru J. Li P. Wang J. Zhou W. Li B. Huang C. (2014). TCMSP: a database of systems pharmacology for drug discovery from herbal medicines. J. Cheminformatics 6, 13. 10.1186/1758-2946-6-13 24735618 PMC4001360

[B39] Seki E. Schwabe R. F. (2015). Hepatic inflammation and fibrosis: functional links and key pathways. Hepatology 61 (3), 1066–1079. 10.1002/hep.27332 25066777 PMC4306641

[B40] Shen B. Lu L. G. (2021a). Efficacy and safety of drugs for nonalcoholic steatohepatitis. J. Dig. Dis. 22 (2), 72–82. 10.1111/1751-2980.12967 33385317

[B41] Shen B. Lu L. G. (2021b). Efficacy and safety of drugs for nonalcoholic steatohepatitis. J. Dig. Dis. 22 (2), 72–82. 10.1111/1751-290.12967 33385317

[B42] Syed-Abdul M. M. (2023a). Lipid metabolism in Metabolic-Associated steatotic liver disease (MASLD). Metabolites 14 (1), 12. 10.3390/metabo1400012 38248815 PMC10818604

[B43] Syed-Abdul M. M. (2023b). Lipid metabolism in Metabolic-Associated steatotic liver disease (MASLD). Metabolites 14 (1), 12. 10.3390/metabo14010012 38248815 PMC10818604

[B44] Szklarczyk D. Morris J. H. Cook H. Kuhn M. Wyder S. Simonovic M. (2017). The STRING database in 2017: quality-controlled protein-protein association networks, made broadly accessible. Nucleic. acids. Res. 45 (D1), D362-D368–D368. 10.1093/nar/gkw937 27924014 PMC5210637

[B45] Terrault N. A. Francoz C. Berenguer M. Charlton M. Heimbach J. (2023). Liver transplantation 2023: status report, Current and future challenges. Clin. Gastroenterol. Hepatol. 21 (8), 2150–2166. 10.1016/j.cgh.2023.04.005 37084928

[B46] Tian W. Liu W. Li J. Yang L. Pan H. (2021). Bioinformatics Study on the regulation of NLRP3 inflammasome activation by Jianpi huayu jiedu formula. Traditional Chin. Drug Res. Clin. Pharmacol. 32 (09), 1321–1328. 10.19378/j.issn.1003-9783.2021.09.012

[B47] Tsuchida T. Lee Y. A. Fujiwara N. Ybanez M. Allen B. Martins S. (2018). A simple diet- and chemical-induced murine NASH model with rapid progression of steatohepatitis, fibrosis and liver cancer. J. Hepatol. 69 (2), 385–395. 10.1016/j.jhep.2018.03.011 29572095 PMC6054570

[B48] Tu C. Qian C. Li S. Lin D. Y. Liu Z. Y. Ouyang W. G. (2025). Targeting the chromatin remodeler BAZ2B mitigates hepatic senescence and MASH fibrosis. Nat. Aging 5, 1063–1078. 10.1038/s43587-025-00862-w 40389730

[B49] Wah K. C. Nik M. N. Mahadeva S. (2017). A randomized trial of silymarin for the treatment of nonalcoholic steatohepatitis. Clin. Gastroenterol. Hepatol. 15 (12), 1940–1949.e8. 10.1016/j.cgh.2017.04.016 28419855

[B50] Xia H. Hui K. M. (2014). Mechanism of cancer drug resistance and the involvement of noncoding RNAs. Curr. Med. Chem. 21 (26), 3029–3041. 10.2174/0929867321666140414101939 24735364

[B51] Xiang M. Liu T. Tan W. Ren H. Li H. Liu J. (2016). Effects of kinsenoside, a potential immunosuppressive drug for autoimmune hepatitis, on dendritic cells/CD8(+) T cells communication in mice. Hepatology 64 (6), 2135–2150. 10.1002/hep.28825 27639182

[B52] Xie K. Peng S. Zheng L. Zhao Z. Gan H. Huang X. (2021). Jianpi huayu jiedu decoction intervenes the autophagy and apoptosis of gastric mucosal epithelial cells in rats with gastric precancerous lesions by regulating PI3K/Akt/HIF-1α pathway. Traditional Chin. Drug Res. Clin. Pharmacol. 32 (10), 1444–1451. 10.19378/j.issn.1003-9783.2021.10.005

[B53] Xu X. Poulsen K. L. Wu L. Liu S. Miyata T. Song Q. (2022a). Targeted therapeutics and novel signaling pathways in non-alcohol-associated fatty liver/steatohepatitis (NAFL/NASH). Signal Transduct. Target. Ther. 7 (1), 287. 10.1038/s41392-022-01119-3 35963848 PMC9376100

[B54] Xu X. Poulsen K. L. Wu L. Liu S. Miyata T. Song Q. (2022b). Targeted therapeutics and novel signaling pathways in non-alcohol-associated fatty liver/steatohepatitis (NAFL/NASH). Signal Transduct. Target. Ther. 7 (1), 287. 10.1038/s41392-02-01119-3 35963848 PMC9376100

[B55] Xuanfei L. Hao C. Zhujun Y. Yanming L. Jianping G. (2017). Imidazoline I2 receptor inhibitor idazoxan regulates the progression of hepatic fibrosis *via* Akt-Nrf2-Smad2/3 signaling pathway. Oncotarget 8 (13), 21015–21030. 10.18632/oncotarget.15472 28423499 PMC5400562

[B56] Yao H. Wang Y. Zhang X. Li P. Shang L. Chen X. (2023). Targeting peroxisomal fatty acid oxidation improves hepatic steatosis and insulin resistance in Obese mice. J. Biol. Chem. 299 (2), 102845. 10.1016/j.jbc.2022.102845 36586435 PMC9898756

[B57] Ye Q. Liu Y. Zhang G. Deng H. Wang X. Tuo L. (2023). Deficiency of gluconeogenic enzyme PCK1 promotes metabolic-associated fatty liver disease through PI3K/AKT/PDGF axis activation in Male mice. Nat. Commun. 14 (1), 1402. 10.1038/s41467-023-37142-3 36918564 PMC10015095

[B58] Younossi Z. Anstee Q. M. Marietti M. Hardy T. Henry L. Eslam M. (2018). Global burden of NAFLD and NASH: trends, predictions, risk factors and prevention. Nat. Rev. Gastroenterol. Hepatol. 15 (1), 11–20. 10.1038/nrgastro.2017.109 28930295

[B59] Zambo V. Simon-Szabo L. Szelenyi P. Kereszturi E. Banhegyi G. Csala M. (2013). Lipotoxicity in the liver. World J. Hepatol. 5 (10), 550–557. 10.4254/wjh.v5.i10.550 24179614 PMC3812457

[B60] Zhang X. Fan L. Wu J. Xu H. Leung W. Y. Fu K. (2019). Macrophage p38α promotes nutritional steatohepatitis through M1 polarization. J. Hepatol. 71 (1), 163–174. 10.1016/j.jhep.2019.03.014 30914267

[B61] Zhao Q. Liu J. Deng H. Ma R. Liao J. Y. Liang H. (2020a). Targeting mitochondria-located circRNA SCAR alleviates NASH *via* reducing mROS output. Cell 183 (1), 76–93. 10.1016/j.cell.2020.08.009 32931733

[B62] Zhao Q. Wei M. Zhang S. Huang Z. Lu B. Ji L. (2020b). The water extract of Sophorae tonkinensis Radix et Rhizoma alleviates non-alcoholic fatty liver disease and its mechanism. Phytomedicine 77, 153270. 10.1016/j.phymed.2020.153270 32702591

[B63] Zhou J. Zheng Q. Chen Z. (2022). The Nrf2 pathway in liver diseases. Front. Cell. Dev. Biol. 10, 826204. 10.3389/fcell.2022.826204 35223849 PMC8866876

