# Supplementary figures and images for: Jianpi-Huayu-Jiedu formula improves metabolic dysfunction-associated steatohepatitis through ameliorateing hepatic oxidative stress and lipid accumulation in vivo and in vitro
Source: Front Pharmacol. 2025 Sep 10;16:1612726. doi: 10.3389/fphar.2025.1612726 (PMC12457829; doi:10.3389/fphar.2025.1612726)

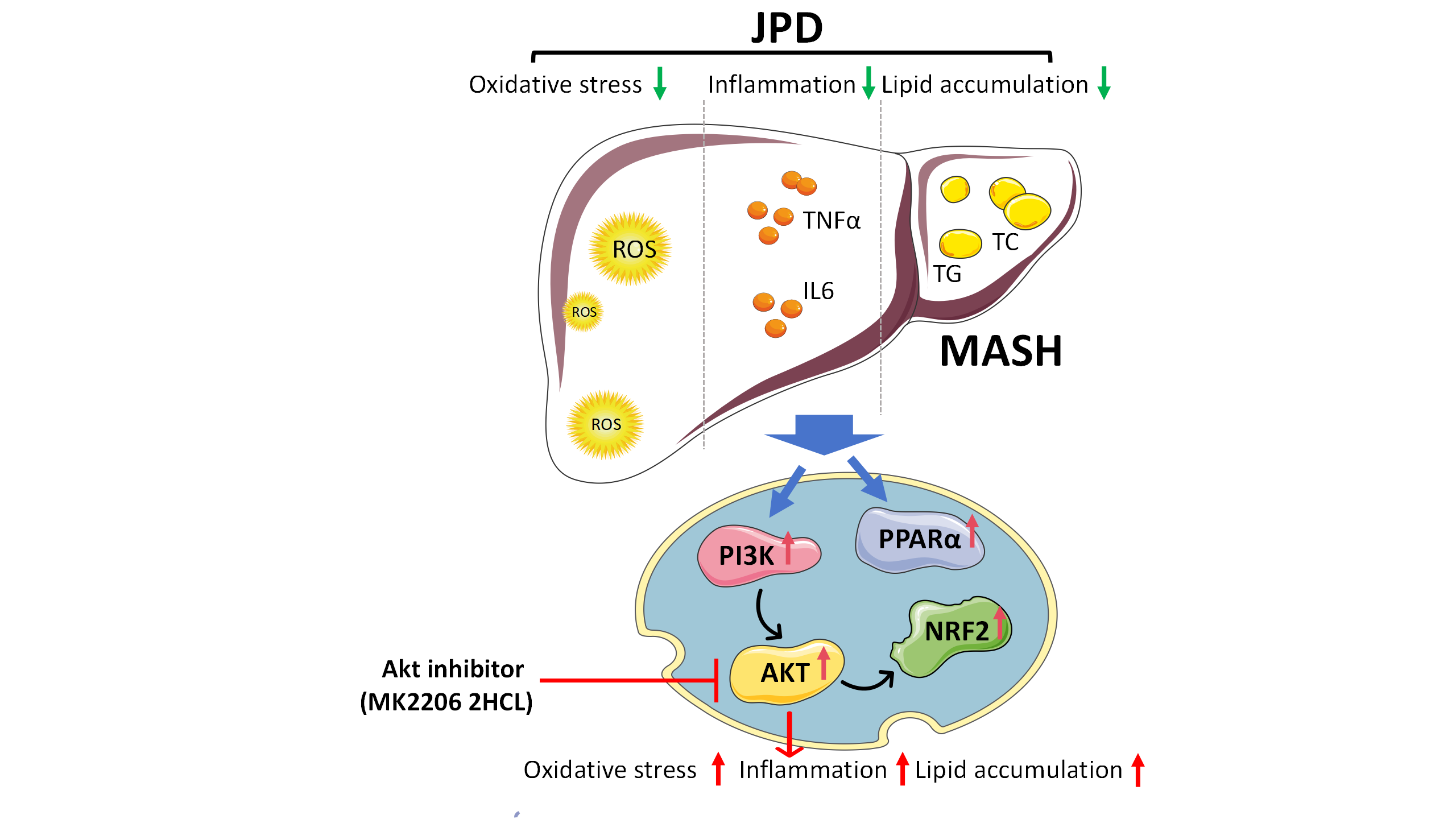

Supplement: Supplementary file 2 [file Image1.tif]
