# Supplementary material for: Jianpi-Huayu-Jiedu formula improves metabolic dysfunction-associated steatohepatitis through ameliorateing hepatic oxidative stress and lipid accumulation in vivo and in vitro
Source: Front Pharmacol. 2025 Sep 10;16:1612726. doi: 10.3389/fphar.2025.1612726 (PMC12457829; doi:10.3389/fphar.2025.1612726)
Supplement: Supplementary file 1 [file Supplementaryfile1.docx]

**Supplementary 1 (Table):**

**Abbreviations:**

| MASH | metabolic dysfunction-associated steatohepatitis |
| --- | --- |
| JPD | Jianpi-Huayu-Jiedu formula |
| MCD | Methionine- and choline-deficient |
| OA&PA | Oleic acid&palmitic acid |
| LPS | Lipopolysaccharide |
| ER | Endoplasmic reticulum |
| ROS | Reactive oxygen species |
| CMM | Chinese Materia Medica |
| CCK-8 | Cell Counting Kit-8 |
| PDB | Protein Data Bank |
| DMEM | Dulbecco's Modified Eagle Medium |
| H&E | Hematoxylin-eosin staining |
| BCA | Bicinchoninic acid |
| DCFDA | 2',7'-dichlorofluorescin diacetate |
| qPCR | Quantitative polymerase chain reaction |
| WB | Western blot analysis |
| cDNA | Complementary DNA |
| PVDF | Polyvinylidene fluoride |
| OB | Oral bioavailability |
| DL | Drug-likeness |
| HCC | Hepatocellular carcinoma |

**Supplementary 2 (Figure):**

**Supplementary 3 (Table):**

**Supplementary material**

| **Table S1: Primer sequences.** | | |
| --- | --- | --- |
| **Gene** | **Forward Primer（5’-3’）** | **Reward Primer（5’-3’）** |
| *IL-1β（human）* | *ATGATGGCTTATTACAGTGGCAA* | *GTCGGAGATTCGTAGCTGGA* |
| *NF-κB（human）* | *AACAGAGAGGATTTCGTTTCCG* | *TTTGACCTGAGGGTAAGACTTCT* |
| *Inos（human）* | *TTCAGTATCACAACCTCAGCAAG* | *TGGACCTGCAAGTTAAAATCCC* |
| *IL-6（human）* | *ACTCACCTCTTCAGAACGAATTG* | *CCATCTTTGGAAGGTTCAGGTTG* |
| *IL-10（human）* | *GACTTTAAGGGTTACCTGGGTTG* | *TCACATGCGCCTTGATGTCTG* |
| *Pparα（human）* | *ATGGTGGACACGGAAAGCC* | *CGATGGATTGCGAAATCTCTTGG* |
| *Fabp4（human）* | *ACTGGGCCAGGAATTTGACG* | *CTCGTGGAAGTGACGCCTT* |
| *Acc （human）* | *ATGTCTGGCTTGCACCTAGTA* | *CCCCAAAGCGAGTAACAAATTCT* |
| *Srebpf1（human）* | *ACAGTGACTTCCCTGGCCTAT* | *GCATGGACGGGTACATCTTCAA* |
| *Srebp1C（human）* | *CGGAACCATCTTGGCAACAGT* | *CGCTTCTCAATGGCGTTGT* |
| *Atp2a2（human）* | *ATGGGGCTCCAACGAGTTAC* | *TTTCCTGCCATACACCCACAA* |
| *Ckb（human）* | *GCTGCGACTTCAGAAGCGA* | *GGCATGAGGTCGTCGATGG* |
| *GAPDH（human）* | *GGAGCGAGATCCCTCCAAAAT* | *GGCTGTTGTCATACTTCTCATGG* |
| *TNF-α （mouse）* | *CCC TCA CAC TCA GAT CAT CTT CT* | *GCT ACG ACG TGG GCT ACA G* |
| *IL-1β（mouse）* | *GCA ACTGTTCCTGAACTCAACT* | *ATCTTTTGGGGTCCGTCAACT* |
| *NF-κB （mouse）* | *ATG GCA GAC GAT GAT CCC TAC* | *TGT TGA CAG TGG TAT TTC TGGTG* |
| *Cd68（mouse）* | *TGTCTGATCTTGCTAGGACCG* | *GAGAGTAACGGCCTTTTTGTGA* |
| *IL-6（mouse）* | *TAGTCCTTCCTACCCCAATTTCC* | *TTGGTCCTTAGCCACTCCTTC* |
| *Inos（mouse）* | *GTT CTC AGC CCA ACA ATA CAA GA* | *GTG GAC GGG TCG ATG TCA C* |
| *Argl（mouse）* | *CTCCAAGCCAAAGTCCTTAGAG* | *AGGAGCTGTCATTAGGGACATC* |
| *Il4（mouse）* | *GGTCTCAACCCCCAGCTAGT* | *GCCGATGATCTCTCTCAAGTGAT* |
| *IL-10（mouse）* | *GCTCTTACTGACTGGCATGAG* | *CGCAGCTCTAGGAGCATGTG* |
| *Ho1（mouse）* | *AAG CCG AGA ATG CTG AGT TCA* | *GCC GTG TAG ATA TGG TAC AAG GA* |
| *Sod2（mouse）* | *CAG ACC TGC CTT ACG ACT ATG G* | *CTC GGT GGC GTT GAG ATT GTT* |
| *Nqo1（mouse）* | *AGGATGGGAGGTACTCGAATC* | *AGGCGTCCTTCCTTATATGCTA* |
| *Nrf2（mouse）* | *TCT TGG AGT AAG TCG AGA AGT GT* | *GTT GAA ACT GAG CGA AAA AGG C* |
| *Gclc（mouse）* | *GGGGTGACGAGGTGGAGTA* | *GTTGGGGTTTGTCCTCTCCC* |
| *Gclm（mouse）* | *AGGAGCTTCGGGACTGTATCC* | *GGGACATGGTGCATTCCAAAA* |
| *Srebp1C（mouse）* | *TGA CCC GGC TAT TCC GTG A* | *CTG GGC TGA GCA ATA CAG TTC* |
| *Acc （mouse）* | *ATG GGC GGA ATG GTC TCT TTC* | *TGG GGA CCT TGT CTT CAT CAT* |
| *Fas（mouse）* | *TATCAAGGAGGCCCATTTTGC* | *TGTTTCCACTTCTAAACCATGCT* |
| *Mgll（mouse）* | *CGGACTTCCAAGTTTTTGTCAGA* | *GCAGCCACTAGGATGGAGATG* |
| *Atgl（mouse）* | *GGATGGCGGCATTTCAGACA* | *CAAAGGGTTGGGTTGGTTCAG* |
| *Shp（mouse）* | *CGACCTGTCACTCTCGGTATC* | *AGGGCTTGGATGATTCTAGTCA* |
| *Pepck（mouse）* | *CTGCATAACGGTCTGGACTTC* | *CAGCAACTGCCCGTACTCC* |
| *Pparα（mouse）* | *AGA GCC CCA TCT GTC CTC TC* | *ACT GGT AGT CTG CAA AAC CAA A* |
| *Acadvl（mouse）* | *CTACTGTGCTTCAGGGACAAC* | *CAAAGGACTTCGATTCTGCCC* |
| *Cpt2（mouse）* | *CAGCACAGCATCGTACCCA* | *TCCCAATGCCGTTCTCAAAAT* |
| *Pgc1α（mouse）* | *TATGGAGTGACATAGAGTGTGCT* | *CCACTTCAATCCACCCAGAAAG* |
| *β-actin（mouse）* | *GGCTGTATTCCCCTCCATCG* | *CCAGTTGGTAACAATGCCATGT* |

**Supplementary 4 (Table):**

**Docking Screening（JPD）**

| Chemical Constituents | m/z | Peak Height（Counts） | Molecular Formula |
| --- | --- | --- | --- |
| Quercetin | 303.0499 | 9872 | C15H10O7 |
| Berberine | 336.1225 | 42371 | C20H18NO4 |
| Pseudoginsenoside F11 | 823.4824/845.4909 | 252784（pos）/3740676（neg） | C47H80O18 |
| Ginsenoside Rb1 | 599.3005 | 8054519 | C54H92O23 |
| Isoallylbenzene | 119.0857 | 335278 | C9H10 |
| Phthalic anhydride | 149.0237 | 569862 | C8H4O3 |
| Purine | 119.0367 | 1156456 | C5H4N4 |
| Phenethylamine | 122.0965 | 242997 | C8H11N |

**Supplementary 5 (Table):**

**The Docking Score**

| MOL | Chemical Constituents | Targets | Binding Energy(kcal/mol) |
| --- | --- | --- | --- |
| MOL000098 | Quercetin | AKT1 | -5.62 |
| MOL001454 | Berberine | AKT1 | -6.9 |
| MOL011438 | Pseudoginsenoside F11 | AKT1 | -11.3 |
| MOL005331 | Ginsenoside Rb1 | AKT1 | -10.5 |
| MOL001030 | Isoallylbenzene | AKT1 | -5.5 |
| MOL008250 | Phthalic anhydride | AKT1 | -6.2 |
| MOL013420 | Purine | AKT1 | -5 |
| MOL002645 | Phenethylamine | AKT1 | -5 |
| MOL000098 | Quercetin | NRF2 | -5.8 |
| MOL001454 | Berberine | NRF2 | -8.8 |
| MOL011438 | Pseudoginsenoside F11 | NRF2 | -11.7 |
| MOL005331 | Ginsenoside Rb1 | NRF2 | -11.1 |
| MOL001030 | Isoallylbenzene | NRF2 | -6 |
| MOL008250 | Phthalic anhydride | NRF2 | -6.3 |
| MOL013420 | Purine | NRF2 | -4.2 |
| MOL002645 | Phenethylamine | NRF2 | -5.3 |
| MOL000098 | Quercetin | PPARα | -5.7 |
| MOL001454 | Berberine | PPARα | -8.4 |
| MOL011438 | Pseudoginsenoside F11 | PPARα | -9.8 |
| MOL005331 | Ginsenoside Rb1 | PPARα | -11 |
| MOL001030 | Isoallylbenzene | PPARα | -5.6 |
| MOL008250 | Phthalic anhydride | PPARα | -6.9 |
| MOL013420 | Purine | PPARα | -5.4 |
| MOL002645 | Phenethylamine | PPARα | -5.3 |

**Supplementary 6 (Figure):**
